# Concise Read-Only Specifications for Better Synthesis of Programs with Pointers

**DOI:** 10.1007/978-3-030-44914-8_6

**Published:** 2020-04-18

**Authors:** Andreea Costea, Amy Zhu, Nadia Polikarpova, Ilya Sergey

**Affiliations:** ETH Zurich, Zurich, Switzerland; 9 School of Computing, National University of Singapore, Singapore, Singapore; 10 University of British Columbia, Vancouver, Canada; 11 University of California, San Diego, USA; 12 Yale-NUS College, Singapore, Singapore

## Abstract

In program synthesis there is a well-known trade-off between *concise* and *strong* specifications: if a specification is too verbose, it might be harder to write than the program; if it is too weak, the synthesised program might not match the user’s intent. In this work we explore the use of annotations for restricting memory access permissions in program synthesis, and show that they can make specifications much stronger while remaining surprisingly concise. Specifically, we enhance Synthetic Separation Logic (SSL), a framework for synthesis of heap-manipulating programs, with the logical mechanism of *read-only borrows*.

We observe that this minimalistic and conservative SSL extension benefits the synthesis in several ways, making it more (a) *expressive* (stronger correctness guarantees are achieved with a modest annotation overhead), (b) *effective* (it produces more concise and easier-to-read programs), (c) *efficient* (faster synthesis), and (d) *robust* (synthesis efficiency is less affected by the choice of the search heuristic). We explain the intuition and provide formal treatment for read-only borrows. We substantiate the claims (a)–(d) by describing our quantitative evaluation of the borrowing-aware synthesis implementation on a series of standard benchmark specifications for various heap-manipulating programs.

### 1 Introduction

Deductive program synthesis is a prominent approach to the generation of correct-by-construction programs from their declarative specifications [14, 23, 29, 33]. With this methodology, one can represent searching for a program satisfying the user-provided constraints as a proof search in a certain logic. Following this idea, it has been recently observed [34] that the synthesis of correct-by-construction *imperative heap-manipulating* programs (in a language similar to C) can be implemented as a proof search in a version of Separation Logic (SL)—a program logic designed for modular verification of programs with pointers [32, 37].

SL-based deductive program synthesis based on *Synthetic* Separation Logic (SSL) [34] requires the programmer to provide a Hoare-style specification for a program of interest. For instance, given the predicate $\mathsf{ls}(x, S)$, which denotes a symbolic heap corresponding to a linked list starting at a pointer `x`, ending with `null`, and containing elements from the set `S`, one can specify the behaviour of the procedure for copying a linked list as follows:

$$\{r \mapsto x * \mathsf{ls}(x, S)\}\ \texttt{listcopy(r)}\ \{r \mapsto y * \mathsf{ls}(x, S) * \mathsf{ls}(y, S)\} \quad (1)$$

* Work done during an internship at NUS School of Computing in Summer 2019.

The precondition of specification (1), defining the shape of the initial heap, is illustrated by the figure above. It requires the heap to contain a pointer `r`, which is taken by the procedure as an argument and whose stored value, `x`, is the head pointer of the list to be copied. The list itself is described by the symbolic heap predicate instance $\mathsf{ls}(\mathtt{x}, \mathtt{S})$, whose footprint is assumed to be *disjoint* from the entry $\mathtt{r} \mapsto \mathtt{x}$, following the standard semantics of the *separating conjunction* operator ($*$) [32]. The postcondition asserts that the final heap, in addition to containing the original list $\mathsf{ls}(\mathtt{x}, \mathtt{S})$, will contain a new list starting from `y` whose contents `S` are the same as of the original list, and also that the pointer `r` will now point to the head `y` of the list copy. Our specification is incomplete: it allows, for example, duplicating or rearranging elements. One hopes that such a program is unlikely to be synthesised. In synthesis, it is common to provide incomplete specs: writing complete ones can be as hard as writing the program itself.

#### 1.1 Correct Programs that Do Strange Things

Provided the definition of the heap predicate ls and the specification (1), the SuSLik tool, an implementation of the SSL-based synthesis [34], will produce the program depicted in Fig. 1. It is easy to check that this program satisfies the ascribed spec (1). Moreover, it correctly duplicates the original list, faithfully preserving its contents and the ordering. However, an astute reader might notice a certain oddity in the way it treats the initial list provided for copying. According to the postcondition of (1), the value of the pointer `r` stored in a local immutable variable `y1` on line `9` is the head of the copy of the original list's tail. Quite unexpectedly, the pointer `y1` becomes the tail of the original list on line `11`, while the *original list's* tail pointer `nxt`, once assigned to `*(y + 1)` on line `13`, becomes the tail of the *copy*!

Indeed, the exercise in tail swapping is totally pointless: not only does it produces less "natural" and readable code, but the resulting program's locality properties are unsatisfactory; for instance, this pro-

```
1  void listcopy (loc r) {
2    let x = *r;
3    if (x == 0) {
4    } else {
5      let v = *x;
6      let nxt = *(x + 1);
7      *r = nxt;
8      listcopy(r);
9      let y1 = *r;
10     let y = malloc(2);
11     *(x + 1) = y1;
12     *r = y;
13     *(y + 1) = nxt;
14     *y = v;
15   } }
```

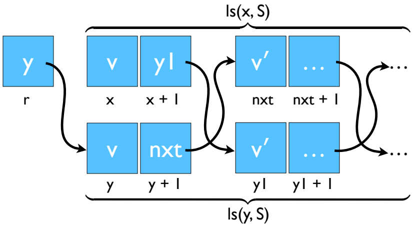

Fig. 1: Result program for spec (1) and the shape of its final heap.

gram cannot be plugged into a concurrent setting where multiple threads rely on $\mathsf{ls}(\mathtt{x}, \mathtt{S})$ to be unchanged.

The issue with the result in Fig. 1 is caused by specification (1) being *too permissive*: it does not prevent the synthesised program from *modifying* the structure of the initial list, while creating its copy. Luckily, the SL community has devised a number of SL extensions that allow one to impose such restrictions, like declaring a part of the provided symbolic heap as *read-only* [5,8,9,11,15,20,21], *i.e.*, forbidden to modify by the specified code.

#### 1.2 Towards Simple Read-Only Specifications for Synthesis

The main challenge of introducing read-only annotations (commonly also referred to as *permissions*)[5] into Separation Logic lies in establishing the discipline for performing sound accounting in the presence of mixed read-only and mutating heap accesses by different components of a program.

As an example, consider a simple symbolic heap $\left\{\mathtt{x} \overset{\mathsf{M}}{\mapsto} \mathtt{f} * \mathtt{r} \overset{\mathsf{M}}{\mapsto} \mathtt{h}\right\}$ that declares two *mutable* (*i.e.*, allowed to be written to) pointers `x` and `r`, that point to unspecified values `f` and `h`, correspondingly. With this symbolic heap, is it safe to call the following function that modifies the contents of `r` but not of `x`?

$$\left\{\mathtt{x} \overset{\mathsf{RO}}{\mapsto} \mathtt{f} * \mathtt{r} \overset{\mathsf{M}}{\mapsto} \mathtt{h}\right\} \ \mathtt{readX(x,\ r)}\ \left\{\mathtt{x} \overset{\mathsf{RO}}{\mapsto} \mathtt{f} * \mathtt{r} \overset{\mathsf{M}}{\mapsto} \mathtt{f}\right\} \tag{2}$$

The precondition of `readX` requires a weaker form of access permission for `x` (read-only, RO), while the considered heap asserts a stronger *write* permission (M). It should be possible to satisfy `readX`'s requirement by providing the necessary read-only permission for `x`. To do so, we need to agree on a discipline to "adapt" the caller's *write*-permission M to the callee's *read-only* permission RO. While seemingly trivial, if implemented naïvely, accounting of RO permissions in SL might compromise either soundness or completeness of the logical reasoning.

A number of proposals for logically sound interplay between write- and read-only access permissions in the presence of function calls has been described in the literature [7–9,11,13,20,30]. Some of these works manage to maintain the simplicity of having only *mutable*/*read-only* annotations when confined to the sequential setting [9,11,13]. More general (but harder to implement) approaches rely on *fractional permissions* [8,25], an expressive mechanism for permission accounting, with primary applications in concurrent reasoning [7,28]. We started this project by attempting to adapt some of those logics [9,11,13] as an extension of SSL in order to reap the benefits of read-only annotations for the synthesis of sequential program. The main obstacle we encountered involved definitions of inductive heap predicates with *mixed* permissions. For instance, how can one specify a program that modifies the contents of a linked list, but not its structure? Even though it seemed possible to enable this treatment of predicates via permission multiplication [25], developing support for this machinery on top of existing SuSLik infrastructure was a daunting task. Therefore, we had to look for a technically simpler solution.

[5] We will be using the words "annotation" and "permission" interchangeably.

#### 1.3 Our Contributions

*Theoretical Contributions.* Our main conceptual innovation is the idea of instrumenting SSL with symbolic *read-only borrows* to enable faster and more predictable program synthesis. Borrows are used to annotate symbolic heaps in specifications, similarly to abstract fractional permissions from the deductive verification tools, such as Chalice and VeriFast [20,21,27]. They enable simple but principled lightweight threading of heap access permissions from the callers to callees and back, while enforcing *read-only* access whenever it is required. For basic intuition on read-only borrows, consider the specification below:

$$\left\{x \overset{a}{\mapsto} f * y \overset{b}{\mapsto} g * r \overset{M}{\mapsto} h\right\} \texttt{readXY(x, y, r)} \left\{x \overset{a}{\mapsto} f * y \overset{b}{\mapsto} g * r \overset{M}{\mapsto} (f + g)\right\} \quad (3)$$

The precondition requires a heap with three pointers, `x`, `y`, and `r`, pointing to unspecified `f`, `g`, and `h`, correspondingly. Both `x` and `y` are going to be treated as read-only, but now, instead of simply annotating them with RO, we add *symbolic borrowing annotations* a and b. The semantics of these borrowing annotations is the same as that of other ghost variables (such as `f`). In particular, the *callee* must behave correctly for any valuation of a and b, which leaves it no choice but to treat the corresponding heap fragments as read-only (hence preventing the heap fragments from being written). On the other hand, from the perspective of the *caller*, they serve as formal parameters that are substituted with actuals of caller's choosing: for instance, when invoked with a caller's symbolic heap $\left\{x \overset{M}{\mapsto} 1 * y \overset{c}{\mapsto} 2 * r \overset{M}{\mapsto} 0\right\}$ (where c denotes a read-only borrow of the caller), `readXY` is guaranteed to "restore" the same access permissions in the postcondition, as per the substitution $[M/a, c/b]$. The example above demonstrates that read-only borrows are straightforward to compose when reasoning about code with function calls. They also make it possible to define *borrow-polymorphic* inductive heap predicates, *e.g.*, enhancing ls from spec (1) so it can be used in specifications with mixed access permissions on their components.[6] Finally, read-only borrows make it almost trivial to adapt the existing SSL-based synthesis to work with read-only access permissions; they reduce the complex permission *accounting* to easy-to-implement permission *substitution.*

*Practical Contributions.* Our first practical contribution is ROBoSuSLik—an enhancement of the SuSLik synthesis tool [34] with support for read-only borrows, which required us to modify less than 100 lines of the original code.

Our second practical contribution is the extensive evaluation of synthesis with read-only permissions, on a standard benchmark suite of specifications for heap-manipulating programs. We compare the behaviour, performance, and the outcomes of the synthesis when run with the standard ("all-mutable") specifications and their analogues instrumented with read-only permissions wherever reasonable. By doing so, we substantiate the following claims regarding the practical impact of using read-only borrows in SSL specifications:

- First, we show that synthesis of read-only specifications is more *efficient*: it does *less backtracking* while searching for a program that satisfies the imposed constraints, entailing better performance.

[6] We will present borrow-polymorphic inductive heap predicates in Sec. 2.4.

– Second, we demonstrate that borrowing-aware synthesis is more *effective*: specifications with read-only annotations lead to more concise and human-readable programs, which do not perform redundant operations.
– Third, we observe that read-only borrows increase *expressivity* of the synthesis: in most of the cases enhanced specifications provide stronger correctness guarantees for the results, at almost no additional annotation overhead.
– Finally, we show that read-only borrows make the synthesis more *robust*: its results and performance are less likely to be affected by the unification order or the order of the attempted rule applications during the search.

*Paper Outline.* We start by showcasing the intricacies and the virtues of SSL-based synthesis with read-only specifications in Sec. 2. We provide the formal account of read-only borrows and present the modified SSL rules, along with the soundness argument in Sec. 3. We report on the implementation and evaluation of the enhanced synthesis in Sec. 4. We conclude with a discussion on the limitations of read-only borrows in Sec. 5 and compare to related work in Sec. 6.

### 2 Program Synthesis with Read-Only Borrows

We introduce the enhancement of SSL with read-only borrows by walking the reader through a series of small but characteristic examples of deductive synthesis with separation logic. We provide the necessary background on SSL in Sec. 2.1; the readers familiar with the logic may want to skip to Sec. 2.2.

#### 2.1 Basics of SSL-based Deductive Program Synthesis

In a deductive Separation Logic-based synthesis, a client provides a specification of a function of interest as a pair of pre- and post-conditions, such as $\{\mathcal{P}\}$ `void foo(loc x, int i)` $\{\mathcal{Q}\}$. The precondition $\mathcal{P}$ constrains the symbolic state necessary to run the function safely (*i.e.*, without crashes), while the postcondition $\mathcal{Q}$ constrains the resulting state at the end of the function's execution. A function body `c` satisfying the provided specification is obtained as a result of deriving the SSL statement, representing the synthesis *goal*:

$$\{\mathtt{x}, \mathtt{i}\}\,;\, \{\mathcal{P}\} \leadsto \{\mathcal{Q}\} \,|\, \mathtt{c}$$

In the statement above, `x` and `i` are *program variables*, and they are explicitly stated in the environment $\Gamma = \{\mathtt{x}, \mathtt{i}\}$. Variables that appear in $\{\mathcal{P}\}$ and that are not program variables are called (logical) *ghost* variables, while the non-program variables that only appear in $\{\mathcal{Q}\}$ are referred to as (logical) *existential* ones (EV). The meaning of the statement $\Gamma; \{\mathcal{P}\} \leadsto \{\mathcal{Q}\} \,|\, \mathtt{c}$ is the *validity* of the Hoare-style triple $\{\mathcal{P}\}\ \mathtt{c}\ \{\mathcal{Q}\}$ for all possible values of variables from $\Gamma$.[7] Both pre- and postcondition contain a *spatial* part describing the shape of the symbolic state (spatial formulae are ranged over via `P`, `Q`, and `R`), and a *pure* part (ranged over via $\phi$, $\psi$, and $\xi$), which states the relations between variables (both program and logical). A derivation of an SSL statement is conducted by applying logical

[7] We often care only about the *existence* of a program $c$ to be synthesised, not its specific shape. In those cases we will be using a shorter statement: $\Gamma; \{\mathcal{P}\} \leadsto \{\mathcal{Q}\}$.

rules, which reduce the initial goal to a trivial one, so it can be solved by one of the *terminal* rules, such as, *e.g.*, the rule Emp shown below:

$$\textsc{Emp}\ \frac{\vdash \phi \Rightarrow \psi}{\Gamma;\ \{\phi; \mathsf{emp}\} \rightsquigarrow \{\psi; \mathsf{emp}\} |\ \mathtt{skip}}$$

That is, Emp requires that (*i*) symbolic heaps in both pre- and post-conditions are empty and (*ii*) that the pure part $\phi$ of the precondition implies the pure part $\psi$ of the postcondition. As the result, Emp “emits” a trivial program `skip`. Some of the SSL rules are aimed at simplifying the goal, bringing it to the shape that can be solved with Emp. For instance, consider the following rules:

$$\textsc{Frame}\ \frac{\mathsf{EV}(\Gamma, \mathcal{P}, \mathcal{Q}) \cap \mathsf{Vars}(\mathtt{R}) = \emptyset \quad \Gamma;\ \{\phi; \mathtt{P}\} \rightsquigarrow \{\psi; \mathtt{Q}\} |\ \mathsf{c}}{\Gamma;\ \{\phi; \mathtt{P} * \mathtt{R}\} \rightsquigarrow \{\psi; \mathtt{Q} * \mathtt{R}\} |\ \mathsf{c}}$$

$$\textsc{UnifyHeaps}\ \frac{[\sigma]\mathtt{R}' = \mathtt{R} \quad \emptyset \neq \mathsf{dom}(\sigma) \subseteq \mathsf{EV}(\Gamma, \mathcal{P}, \mathcal{Q}) \quad \Gamma;\ \{\phi; \mathtt{P} * \mathtt{R}\} \rightsquigarrow [\sigma]\{\psi; \mathtt{Q} * \mathtt{R}'\} |\ \mathsf{c}}{\Gamma;\ \{\phi; \mathtt{P} * \mathtt{R}\} \rightsquigarrow \{\psi; \mathtt{Q} * \mathtt{R}'\} |\ \mathsf{c}}$$

Neither of the rules Frame and UnifyHeaps “adds” to the program c being synthesised. However, Frame reduces the goal by removing a matching part `R` (*a.k.a. frame*) from both the pre- and the post-condition. UnifyHeaps non-deterministically picks a substitution $\sigma$, which replaces existential variables in a sub-heap $\mathtt{R}'$ of the postcondition to match the corresponding symbolic heap `R` in the precondition. Both of these rules make choices with regard to what frame `R` to remove or which substitution $\sigma$ to adopt—a point that will be of importance for the development described in Sec. 2.2.

Finally, the following (simplified) rule for producing a *write* command is *operational*, as it emits a part of the program to be synthesised, while also modifying the goal accordingly. The resulting program will, thus, consist of the emitted store $*\mathtt{x} = \mathsf{e}$ of an expression e to the pointer variable `x`. The remainder is synthesised by solving the sub-goal produced by applying the Write rule.

$$\textsc{Write}\ \frac{\mathsf{Vars}(\mathsf{e}) \subseteq \Gamma \quad \mathsf{e} \neq \mathsf{e}' \quad \Gamma;\ \{\phi; \mathtt{x} \mapsto \mathsf{e} * \mathtt{P}\} \rightsquigarrow \{\psi; \mathtt{x} \mapsto \mathsf{e} * \mathtt{Q}\} |\ \mathsf{c}}{\Gamma;\ \{\phi; \mathtt{x} \mapsto \mathsf{e}' * \mathtt{P}\} \rightsquigarrow \{\psi; \mathtt{x} \mapsto \mathsf{e} * \mathtt{Q}\} |\ *\mathtt{x} = \mathsf{e}; \mathsf{c}}$$

As it is common with proof search, should no rule apply to an intermediate goal within one of the derivations, the deductive synthesis back-tracks, possibly discarding a partially synthesised program fragment, trying alternative derivation branches. For instance, firing UnifyHeaps to unify wrong sub-heaps might lead the search down a path to an unsatisfiable goal, eventually making the synthesis back-track and leading to longer search. Consider also a misguided application of Write into a certain location, which can cause the synthesizer to generate a less intuitive program that “makes up” for the earlier spurious writes. This is precisely what we are going to fix by introducing read-only annotations.

### 2.2 Reducing Non-Determinism with Read-Only Annotations

Consider the following example adapted from the original SSL paper [34]. While the example is intentionally artificial, it captures a frequent synthesis scenario—non-determinism during synthesis. This specification allows a certain degree of freedom in how it can be satisfied:

$$\{\texttt{x} \mapsto 239 * \texttt{y} \mapsto 30\}\ \texttt{void pick(loc x, loc y)}\ \{\texttt{z} \leq 100; \texttt{x} \mapsto \texttt{z} * \texttt{y} \mapsto \texttt{z}\} \qquad (4)$$

It seems logical for the synthesis to start the program derivation by applying the rule UnifyHeaps, thus reducing the initial goal to the one of the form

$$\{\texttt{x}, \texttt{y}\}\,; \{\texttt{x} \mapsto 239 * \texttt{y} \mapsto 30\} \rightsquigarrow \{239 \leq 100; \texttt{x} \mapsto 239 * \texttt{y} \mapsto 239\}$$

This new goal has been obtained by picking one particular substitution $\sigma = [239/\texttt{z}]$ (out of multiple possible ones), which delivers two identical *heaplets* of the form $\texttt{x} \mapsto 239$ in pre- and postcondition. It is time for the Write rule to strike to fix the discrepancy between the symbolic heap in the pre- and postcondition by emitting the command $*\texttt{y} = 239$ (at last, some executable code!), and resulting in the following new goal (notice the change of $y$-related entry in the precondition):

$$\{\texttt{x}, \texttt{y}\}\,; \{\texttt{x} \mapsto 239 * \texttt{y} \mapsto 239\} \rightsquigarrow \{239 \leq 100; \texttt{x} \mapsto 239 * \texttt{y} \mapsto 239\}$$

What follows are two applications of the Frame rule to the common symbolic heaps, leading to the goal: $\{\texttt{x}, \texttt{y}\}\ \{\textsf{emp}\} \rightsquigarrow \{239 \leq 100; \textsf{emp}\}$. At this point, we are clearly in trouble. The pure part of the precondition is simply $\textsf{true}$, while the postcondition's pure part is $239 \leq 100$, which is unsolvable.

Turns out that our initial pick of the substitution $\sigma = [239/\texttt{z}]$ was an unfortunate one, and we should discard the series of rule applications that followed it, back-track and adopt a different substitution, *e.g.*, $\sigma' = [30/\texttt{z}]$, which will indeed result in solving our initial goal.[8]

Let us now consider the same specification for `pick` that has been enhanced by explicitly annotating parts of the symbolic heap as mutable and read-only:

$$\left\{\texttt{x} \overset{\textsf{M}}{\mapsto} 239 * \texttt{y} \overset{\textsf{RO}}{\mapsto} 30\right\}\ \texttt{void pick(loc x, loc y)}\ \left\{\texttt{z} \leq 100; \texttt{x} \overset{\textsf{M}}{\mapsto} \texttt{z} * \texttt{y} \overset{\textsf{RO}}{\mapsto} \texttt{z}\right\} \qquad (5)$$

In this version of SSL, the effect of rules such as Emp, Frame, and UnifyHeaps remains the same, while operational rules such as Write, become *annotation-aware*. Specifically, the rule Write is now replaced by the following one:

$$\textsc{WriteRO}\ \frac{\textsf{Vars}(\texttt{e}) \subseteq \Gamma \qquad \texttt{e} \neq \texttt{e}' \qquad \Gamma; \left\{\phi; \texttt{x} \overset{\textsf{M}}{\mapsto} \texttt{e} * \texttt{P}\right\} \rightsquigarrow \left\{\psi; \texttt{x} \overset{\textsf{M}}{\mapsto} \texttt{e} * \texttt{Q}\right\} \Big| \ \texttt{c}}{\Gamma; \left\{\phi; \texttt{x} \overset{\textsf{M}}{\mapsto} \texttt{e}' * \texttt{P}\right\} \rightsquigarrow \left\{\psi; \texttt{x} \overset{\textsf{M}}{\mapsto} \texttt{e} * \texttt{Q}\right\} \Big| \ *\texttt{x} = \texttt{e}; \texttt{c}}$$

Notice how in the rule above the heaplets of the form $\texttt{x} \overset{\textsf{M}}{\mapsto} \texttt{e}$ are now annotated with the access permission $\textsf{M}$, which explicitly indicates that the code may modify the corresponding heap location.

Following with the example specification (5), we can imagine a similar scenario when the rule UnifyHeaps picks the substitution $\sigma = [239/\texttt{z}]$. Should this be the case, the next application of the rule WriteRO will not be possible, due to the *read-only* annotation on the heaplet $\texttt{y} \overset{\textsf{RO}}{\mapsto} 239$ in the resulting sub-goal:

$$\{\texttt{x}, \texttt{y}\}\,; \left\{\texttt{x} \overset{\textsf{M}}{\mapsto} 239 * \ \boxed{\texttt{y} \overset{\textsf{RO}}{\mapsto} 30}\right\} \rightsquigarrow \left\{\texttt{z} \leq 100; \texttt{x} \overset{\textsf{M}}{\mapsto} 239 * \ \boxed{\texttt{y} \overset{\textsf{RO}}{\mapsto} 239}\right\}$$

As the $\textsf{RO}$ access permission prevents the synthesised code from modifying the greyed heaplets, the synthesis search is forced to back-track, picking an alternative substitution $\sigma'=[30/\texttt{z}]$ and converging on the desirable program $*\texttt{x}=30$.

[8] One might argue that it was possible to detect the unsolvable conjunct $239 \leq 100$ in the postcondition immediately after performing substitution, thus sparing the need to proceed with this derivation further. This is, indeed, a possibility, but in general it is hard to argue which of the heuristics in applying the rules will work better in general. We defer the quantitative argument on this matter until Sec. 4.4.

#### 2.3 Composing Read-Only Borrows

Having synthesised the `pick` function from specification (5), we would like to use it in future programs. For example, imagine that at some point, while synthesising another program, we see the following as an intermediate goal:

$$\{\mathtt{u}, \mathtt{v}\}\,;\left\{\mathtt{u} \overset{\mathsf{M}}{\mapsto} 239 * \mathtt{v} \overset{\mathsf{M}}{\mapsto} 30 * \mathtt{P}\right\} \leadsto \left\{\mathtt{w} \leq 200; \mathtt{u} \overset{\mathsf{M}}{\mapsto} \mathtt{w} * \mathtt{v} \overset{\mathsf{M}}{\mapsto} \mathtt{w} * \mathtt{Q}\right\} \qquad (6)$$

It is clear that, modulo the names of the variables, we can synthesise a part of the desired program by emitting a call $\mathtt{pick}(\mathtt{u}, \mathtt{v})$, which we can then reduce to the goal $\{\mathtt{u}, \mathtt{v}\}$ $\{\mathtt{P}\} \leadsto \{\mathtt{w} \leq 200; \mathtt{Q}\}$ via an application of Frame.

Why is emitting such a call to $\mathtt{pick}()$ safe? Intuitively, this can be done because the precondition of the spec (5) is *weaker* than the one in the goal (6). Indeed, the precondition of the latter provides the full (mutable) access permission on the heap portion $\mathtt{v} \overset{\mathsf{M}}{\mapsto} 30$, while the pre/postcondition of former requires a weaker form of access, namely read-only: $\mathtt{y} \overset{\mathsf{RO}}{\mapsto} 30$. Therefore, our logical foundations should allow temporary "downgrading" of an access permission, *e.g.*, from $\mathsf{M}$ to $\mathsf{RO}$, for the sake of synthesising calls. While allowing this is straightforward and can be done similarly to up-casting a type in languages like Java, what turns out to be less trivial is making sure that the caller's initial stronger access permission ($\mathsf{M}$) is *restored* once $\mathtt{pick}(\mathtt{u}, \mathtt{v})$ returns.

*Non-solutions.* Perhaps, the simplest way to allow the call to a function with a weaker (in terms of access permissions) specification, would be to (a) downgrade the caller's permissions on the corresponding heap fragments to $\mathsf{RO}$, and (b) recover the permissions as per the callee's specification. This approach significantly reduces the expressivity of the logic (and, as a consequence, completeness of the synthesis). For instance, adopting this strategy for using specification (5) in the goal (6) would result in the unsolvable sub-goal of the form $\{\mathtt{u}, \mathtt{v}\}\,;\left\{\mathtt{u} \overset{\mathsf{M}}{\mapsto} 30 * \mathtt{v} \overset{\mathsf{RO}}{\mapsto} 30 * \mathtt{P}\right\} \leadsto \left\{\mathtt{u} \overset{\mathsf{M}}{\mapsto} 30 * \mathtt{v} \overset{\mathsf{M}}{\mapsto} 30 * \mathtt{Q}\right\}$. This is due to the fact that the postcondition requires the heaplet $\mathtt{v} \overset{\mathsf{M}}{\mapsto} 30$ to have the write-permission $\mathsf{M}$, while the new precondition only provides the $\mathsf{RO}$-access.

Another way to cater for a weaker callee's specification would be to "chip out" a $\mathsf{RO}$-permission from a caller's $\mathsf{M}$-annotation (in the spirit of fractional permissions), offer it to the callee, and then "merge" it back to the caller's full-blown permission upon return. This solution works for simple examples, but not for heap predicates with mixed permissions (discussion in Sec. 6). Yet another approach would be to create a "$\mathsf{RO}$ clone" of the caller's $\mathsf{M}$-annotation, introducing an axiom of the form $\mathtt{x} \overset{\mathsf{M}}{\mapsto} \mathtt{t} \dashv\vdash \mathtt{x} \overset{\mathsf{M}}{\mapsto} \mathtt{t} * \mathtt{x} \overset{\mathsf{RO}}{\mapsto} \mathtt{t}$. The created component $\mathtt{x} \overset{\mathsf{RO}}{\mapsto} \mathtt{t}$ could be provided to the callee and discarded upon return since the caller retained the full permission of the original heap. Several works on $\mathsf{RO}$ permissions have adopted this approach [9, 11, 13]. While discarding such clones works just fine for sequential program verification, in the case of synthesis guided by pre- and postconditions, *incomplete* postconditions could lead to intractable goals.

*Our solution.* The key to gaining the necessary expressivity *wrt.* passing/returning access permissions, while maintaining a sound yet simple logic, is *treating access permissions as first-class values.* A natural consequence of this treatment is that immutability annotations can be symbolic (*i.e.*, variables of a special sort

"permission"), and the semantics of such variables is well understood; we refer to these symbolic annotations as *read-only borrows*.[9] For instance, using borrows, we can represent the specification (5) as an equivalent one:

$$\left\{\mathtt{x} \overset{\mathsf{M}}{\mapsto} 239 * \mathtt{y} \overset{\mathsf{a}}{\mapsto} 30\right\} \ \mathtt{void\ pick(loc\ x,\ loc\ y)} \ \left\{\mathtt{z} \leq 100; \mathtt{x} \overset{\mathsf{M}}{\mapsto} \mathtt{z} * \mathtt{y} \overset{\mathsf{a}}{\mapsto} \mathtt{z}\right\} \qquad (7)$$

The only substantial difference with spec (5) is that now the pointer y's access permission is given an *explicit name* a. Such named annotations (*a.k.a.* borrows) are treated as RO by the callee, as long as the pure precondition does not constrain them to be mutable. However, giving these permissions names achieves an important goal: performing accurate accounting while composing specifications with different access permissions. Specifically, we can now emit a call to pick(u, v) as specified by (7) from the goal (6), keeping in mind the substitution $\sigma = [\mathtt{u}/\mathtt{x}, \mathtt{v}/\mathtt{y}, \mathsf{M}/\mathsf{a}]$. This call now accounts for borrows as well, and makes it straightforward to restore v's original permission M upon returning.

Following the same idea, borrows can be naturally composed through capture-avoiding substitutions. For instance, the same specification (7) of pick could be used to advance the following modified version of the goal (6):

$$\{\mathtt{u}, \mathtt{v}\}\,;\left\{\mathtt{u} \overset{\mathsf{M}}{\mapsto} 239 * \mathtt{v} \overset{\mathsf{c}}{\mapsto} 30 * \mathtt{P}\right\} \rightsquigarrow \left\{\mathtt{w} \leq 210; \mathtt{u} \overset{\mathsf{M}}{\mapsto} \mathtt{w} * \mathtt{v} \overset{\mathsf{c}}{\mapsto} \mathtt{w} * \mathtt{Q}\right\}$$

by means of taking the substitution $\sigma' = [\mathtt{u}/\mathtt{x}, \mathtt{v}/\mathtt{y}, \mathsf{c}/\mathsf{a}]$.

#### 2.4 Borrow-Polymorphic Inductive Predicates

Separation Logic owes its glory to the extensive use of *inductive heap predicates*—a compact way to capture the shape and the properties of finite heap fragments corresponding to recursive linked data structures. Below we provide one of the most widely-used SL predicates, defining the shape of a heap containing a null-terminated singly-linked list with elements from a set S:

$$\begin{array}{rcl} \mathsf{ls}(\mathtt{x}, \mathtt{S}) & \triangleq & \mathtt{x} = 0 \wedge \{\mathtt{S} = \emptyset; \mathsf{emp}\} \\ & | & \mathtt{x} \neq 0 \wedge \{\mathtt{S} = \{\mathtt{v}\} \cup \mathtt{S}_1; [\mathtt{x}, 2] * \mathtt{x} \mapsto \mathtt{v} * \langle \mathtt{x}, 1\rangle \mapsto nxt * \mathsf{ls}(nxt, \mathtt{S}_1)\} \end{array} \qquad (8)$$

The predicate contains two clauses describing the corresponding cases of the list's shape depending on the value of the head pointer x. If x is zero, the list's heap representation is empty, and so is the set of elements S. Alternatively, if x is not zero, it stores a record with two items (indicated by the *block assertion* $[\mathtt{x}, 2]$), such that the *payload* pointer x contains the value v (where $\mathtt{S} = \{\mathtt{v}\} \cup \mathtt{S}_1$ for some set $\mathtt{S}_1$), and the pointer, corresponding to $\mathtt{x} + 1$ (denoted as $\langle \mathtt{x}, 1\rangle$) contains the address of the list's tail, *nxt*.

While expressive enough to specify and enable synthesis of various list-traversing and list-generating recursive functions via SSL, the definition (8) does not allow one to restrict the access permissions to different components of the list: all of the involved memory locations can be mutated (which explains the synthesis issue we described in Sec. 1.1). To remedy this weakness of the traditional SL-style predicates, we propose to *parameterise* them with read-only borrows, thus making them aware of different access permissions to their various components. For instance, we propose to redefine the linked list predicate as follows:

[9] In this regard, our symbolic borrows are very similar to abstract fractional permissions in CHALICE and VERIFAST [21,27]. We discuss the relation in detail in Sec. 6.

$$\mathsf{ls}(\mathtt{x}, \mathtt{S}, \mathsf{a}, \mathsf{b}, \mathsf{c}) \triangleq \mathtt{x} = 0 \land \{\mathtt{S} = \emptyset; \mathsf{emp}\}$$
$$\mid \mathtt{x} \neq 0 \land \left\{\mathtt{S} = \{\mathtt{v}\} \cup \mathtt{S}_1; [\mathtt{x}, 2]^{\mathsf{a}} * \mathtt{x} \overset{\mathsf{b}}{\mapsto} \mathtt{v} * \langle \mathtt{x}, 1\rangle \overset{\mathsf{c}}{\mapsto} nxt * \mathsf{ls}(nxt, \mathtt{S}_1, \mathsf{a}, \mathsf{b}, \mathsf{c})\right\} \quad (9)$$

The new definition (9) is similar to the old one (8), but now, in addition to the standard predicate parameters (*i.e.*, the head pointer x and the set S in this case), also features three borrow parameters a, b, and c that stand as placeholders for the access permissions to some particular components of the list. Specifically, the symbolic borrows b and c control the permissions to manipulate the pointers x and $\mathtt{x}+1$, correspondingly. The borrow a, modifying a block-type heaplet, determines whether the record starting at x can be deallocated with free(x). All the three borrows are passed in the same configuration to the recursive instance of the predicate, thereby imposing the same constraints on the rest of the corresponding list components.

Let us see the borrow-polymorphic inductive predicates in action. Consider the following specification that asks for a function taking a list of arbitrary values and replacing all of them with zeroes:[10]

$$\{\mathsf{ls}(\mathtt{x}, \mathtt{S}, \mathsf{d}, \mathsf{M}, \mathsf{e})\}\ \texttt{void reset(loc x)}\ \{\mathsf{ls}(\mathtt{x}, \mathbb{O}, \mathsf{d}, \mathsf{M}, \mathsf{e})\} \quad (10)$$

The spec (10) gives very little freedom to the function that would satisfy it with regard to permissions to manipulate the contents of the heap, constrained by the predicate $\mathsf{ls}(\mathtt{x}, S, \mathsf{d}, \mathsf{M}, \mathsf{e})$. As the first and the third borrow parameters are instantiated with read-only borrows (d and e), the desired function is not going to be able to change the structural pointers or deallocate parts of the list. The only allowed manipulation is, thus, changing the values of the payload pointers.

This concise specification is pleasantly strong. To wit, in plain SSL, a similar spec (without read-only annotations) would also admit an implementation that fully deallocates the list or arbitrarily changes its length. In order to avoid these outcomes, one would, therefore, need to provide an alternative definition of the predicate ls, which would incorporate the length property too.

Imagine now that one would like to use the implementation of reset satisfying specification (10) to generate a function with the following spec, providing stronger access permissions for the list components:

$$\{\mathsf{ls}(\mathtt{y}, \mathtt{S}, \mathsf{M}, \mathsf{M}, \mathsf{M})\}\ \texttt{void call\_reset(loc y)}\ \{\mathsf{ls}(\mathtt{y}, \mathbb{O}, \mathsf{M}, \mathsf{M}, \mathsf{M})\}$$

During the synthesis of call_reset, a call to reset is generated. For this purpose the access permissions are borrowed and recovered as per spec (10) via the substitution $[\mathtt{y}/\mathtt{x}, \mathsf{M}/\mathsf{d}, \mathsf{M}/\mathsf{e}]$ in a way described in Sec. 2.3.

#### 2.5 Putting It All Together

We conclude this overview by explaining how synthesis via SSL enhanced with read-only borrows avoids the issue with spurious writes outlined in Sec. 1.1.

To begin, we change the specification to the following one, which makes use of the new list predicate (9) and prevents any modifications in the original list.

$$\left\{\mathtt{r} \overset{\mathsf{M}}{\mapsto} \mathtt{x} * \mathsf{ls}(\mathtt{x}, \mathtt{S}, \mathsf{a}, \mathsf{b}, \mathsf{c})\right\}\ \texttt{listcopy(r)}\ \left\{\mathtt{r} \overset{\mathsf{M}}{\mapsto} \mathtt{y} * \mathsf{ls}(\mathtt{x}, \mathtt{S}, \mathsf{a}, \mathsf{b}, \mathsf{c}) * \mathsf{ls}(\mathtt{y}, \mathtt{S}, \mathsf{M}, \mathsf{M}, \mathsf{M})\right\}$$

We should remark that, contrary to the solution sketched at the end of Sec. 1.1, which suggested using the predicate instance of the shape $\mathsf{ls}(\mathtt{x}, \mathtt{S})[\mathsf{RO}]$, our concrete proposal does not allow us to constrain the entire predicate with a single

[10] We use $\mathbb{O}$ as a notation for a multi-set with an arbitrary finite number of zeroes.

| | | | |
|---|---|---|---|
| Variable | $x, y$ | | Alpha-numeric identifiers |
| Size, offset | $n, \iota$ | | Non-negative integers |
| Expression | $e$ | $::=$ | $0 \mid \mathsf{true} \mid x \mid e = e \mid e \wedge e \mid \neg e$ |
| Command | $c$ | $::=$ | $\textbf{let}\ \ x = *(x + \iota) \mid *(x + \iota) = e \mid \textbf{let}\ \ x = \mathtt{malloc}(n) \mid \mathtt{free}(x)$ |
| | | | $\mid \mathtt{err} \mid f(\overline{e_i}) \mid c; c \mid \textbf{if}\ (e)\ \{c\}\ \textbf{else}\ \{c\}$ |
| Fun. dict. | $\Delta$ | $::=$ | $\epsilon \mid \Delta, f\ (\overline{x_i})\ \{\ c\ \}$ |

Fig. 2: Programming language grammar.

| | | | | | | |
|---|---|---|---|---|---|---|
| Pure term | $\phi, \psi, \chi, \alpha$ | $::= 0 \mid \mathsf{true} \mid \mathsf{M} \mid \mathsf{RO} \mid x \mid \phi = \phi \mid \phi \wedge \phi \mid \neg\phi$ | | | | |
| Symbolic heap | $\mathtt{P}, \mathtt{Q}, \mathtt{R}$ | $::= \mathsf{emp} \mid \langle \mathsf{e}, \iota \rangle \overset{\alpha}{\mapsto} \mathsf{e} \mid [\mathsf{e}, \iota]^{\alpha} \mid \mathsf{p}(\overline{\phi_i}) \mid \mathtt{P} * \mathtt{Q}$ | | | | |
| Heap predicate | $\mathcal{D}$ | $::= \mathsf{p}(\overline{\mathtt{x_i}})\ \overline{\langle \mathsf{e_k}, \{\chi_k, \mathtt{R_k}\} \rangle}$ | | | | |
| Function spec | $\mathcal{F}$ | $::= \mathtt{f}(\overline{\mathtt{x_i}}) : \{\mathcal{P}\}\{\mathcal{Q}\}$ | Assertion | $\mathcal{P}, \mathcal{Q}$ | $::=$ | $\{\phi; \mathtt{P}\}$ |
| Environment | $\Gamma$ | $:= \epsilon \mid \Gamma, \mathtt{x}$ | Context | $\Sigma$ | $:=$ | $\epsilon \mid \Sigma, \mathcal{D} \mid \Sigma, \mathcal{F}$ |

Fig. 3: BoSSL assertion syntax.

access permission (*e.g.*, RO). Instead, we allow *fine-grained* access control to its particular elementary components by annotating each one with an individual borrow. The specification above allows the greatest flexibility *wrt.* access permissions to the original list by giving them different names (a, b, c).

In the process of synthesising the non-trivial branch of `listcopy`, the search at some point will come up with the following intermediate goal:

$$\{\mathtt{x}, \mathtt{r}, \mathtt{nxt}, \mathtt{v}, \mathtt{y12}\}\,;$$
$$\left\{\mathtt{S} = \{\mathtt{v}\} \cup \mathtt{S_1}; \mathtt{r} \overset{\mathsf{M}}{\mapsto} \mathtt{y12} * [\mathtt{x}, 2]^{\mathsf{a}} * \mathtt{x} \overset{\mathsf{b}}{\mapsto} \mathtt{v} * \langle \mathtt{x}, 1 \rangle \overset{\mathsf{c}}{\mapsto} \mathtt{nxt} * \mathsf{ls}(\mathtt{y12}, \mathtt{S_1}, \mathsf{M}, \mathsf{M}, \mathsf{M}) * \ldots\right\}$$
$$\rightsquigarrow \left\{[\mathtt{z}, 2]^{\mathsf{M}} * \mathtt{z} \overset{\mathsf{M}}{\mapsto} \mathtt{v} * \langle \mathtt{z}, 1 \rangle \overset{\mathsf{M}}{\mapsto} \mathtt{y12} * \mathsf{ls}(\mathtt{y12}, \mathtt{S_1}, \mathsf{M}, \mathsf{M}, \mathsf{M}) * \ldots\right\}$$

Since the logical variable `z` in the postcondition is an existential one, the greyed part of the symbolic heap can be satisfied by either (a) re-purposing the greyed part of the precondition (which is what the implementation in Sec. 1.1 does), or (b) allocating a corresponding record of two elements (as should be done). With the read-only borrows in place, the unification of the two greyed fragments in the pre- and postcondition via UnifyHeaps fails, because the mutable annotation of $\mathtt{z} \overset{\mathsf{M}}{\mapsto} \mathtt{v}$ in the post cannot be matched by the read-only borrow $\mathtt{x} \overset{\mathsf{b}}{\mapsto} \mathtt{v}$ in the precondition. Therefore, not being able to follow the derivation path (a), the synthesiser is forced to explore an alternative one, eventually deriving the version of `listcopy` without tail-swapping.

### 3 BoSSL: Borrowing Synthetic Separation Logic

We now give a formal presentation of BoSSL—a version of SSL extended with read-only borrows. Fig. 2 and Fig. 3 present its programming and assertion language, respectively. For simplicity, we formalise a core language without theories (*e.g.*, natural numbers), similar to the one of Smallfoot [6]; the only sorts in the core language are locations, booleans, and permissions (where permissions appear only in specifications) and the pure logic only has equality. In contrast, our implementation supports integers and sets (where the latter also only appear in specifications), with linear arithmetic and standard set operations. We do

$$\textsc{Write}\quad \frac{\mathsf{Vars}(\mathtt{e}) \subseteq \Gamma \qquad \mathtt{e} \neq \mathtt{e}' \qquad \Gamma; \left\{\phi; \langle \mathtt{x}, \iota\rangle \overset{\mathsf{M}}{\mapsto} \mathtt{e} * \mathtt{P}\right\} \rightsquigarrow \left\{\psi; \langle \mathtt{x}, \iota\rangle \overset{\mathsf{M}}{\mapsto} \mathtt{e} * \mathtt{Q}\right\} \Big| \, \mathtt{c}}{\Gamma; \left\{\phi; \langle \mathtt{x}, \iota\rangle \overset{\mathsf{M}}{\mapsto} \mathtt{e}' * \mathtt{P}\right\} \rightsquigarrow \left\{\psi; \langle \mathtt{x}, \iota\rangle \overset{\mathsf{M}}{\mapsto} \mathtt{e} * \mathtt{Q}\right\} \Big| * (\mathtt{x} + \iota) = \mathtt{e}; \mathtt{c}}$$

$$\textsc{Alloc}\quad \frac{\begin{array}{c}\mathtt{R} = [\mathtt{z}, \mathtt{n}]^{\alpha} * \mathop{\ast}_{0 \leq \mathtt{i} < \mathtt{n}} \left(\langle \mathtt{z}, \mathtt{i}\rangle \overset{\alpha_{\mathtt{i}}}{\mapsto} \mathtt{e_i}\right) \qquad (\{\mathtt{y}\} \cup \{\overline{\mathtt{t_i}}\}) \cap \mathsf{Vars}(\Gamma, \mathcal{P}, \mathcal{Q}) = \emptyset \qquad \mathtt{z} \in \mathsf{EV}(\Gamma, \mathcal{P}, \mathcal{Q}) \\ \mathtt{R}' \triangleq [\mathtt{y}, \mathtt{n}]^{\mathsf{M}} * \mathop{\ast}_{0 \leq \mathtt{i} < \mathtt{n}} \left(\langle \mathtt{y}, \mathtt{i}\rangle \overset{\mathsf{M}}{\mapsto} \mathtt{t_i}\right) \qquad \Sigma; \Gamma; \{\phi; \mathtt{P} * \mathtt{R}'\} \rightsquigarrow \{\psi; \mathtt{Q} * \mathtt{R}\} \big| \, \mathtt{c}\end{array}}{\Sigma; \Gamma; \{\phi; \mathtt{P}\} \rightsquigarrow \{\psi; \mathtt{Q} * \mathtt{R}\} \big| \, \mathbf{let}\ \mathtt{y} = \mathtt{malloc}(\mathtt{n}); \mathtt{c}}$$

$$\textsc{Free}\quad \frac{\mathtt{R} = [\mathtt{x}, \mathtt{n}]^{\mathsf{M}} * \mathop{\ast}_{0 \leq \mathtt{i} < \mathtt{n}} \left(\langle \mathtt{x}, \mathtt{i}\rangle \overset{\mathsf{M}}{\mapsto} \mathtt{e_i}\right) \qquad \mathsf{Vars}(\{\mathtt{x}\} \cup \{\overline{\mathtt{e_i}}\}) \subseteq \Gamma \qquad \Sigma; \Gamma; \{\phi; \mathtt{P}\} \rightsquigarrow \{\mathcal{Q}\} \big| \, \mathtt{c}}{\Sigma; \Gamma; \{\phi; \mathtt{P} * \mathtt{R}\} \rightsquigarrow \{\mathcal{Q}\} \big| \, \mathtt{free}(\mathtt{x}); \mathtt{c}}$$

Fig. 4: BoSSL derivation rules.

not formalise sort-checking of formulae; however, for readability, we will use the meta-variable $\alpha$ where the intended sort of the pure logic term is "permission", and `Perm` for the set of all permissions. The permission to allocate or deallocate a memory-block $[\mathtt{x}, \mathtt{n}]^{\alpha}$ is controlled by $\alpha$.

#### 3.1 BoSSL rules

New rules of BoSSL are shown in Fig. 4. The figure contains only 3 rules: this minimal adjustment is possible thanks to our approach to unification and permission accounting from first principles. Writing to a memory location requires its corresponding symbolic heap to be annotated as mutable. Note that for a precondition $\left\{\mathsf{a} = \mathsf{M}; \langle \mathtt{x}\rangle \overset{\mathsf{a}}{\mapsto} 5\right\}$, a normalisation rule like SubstLeft would first transform it into $\left\{\mathsf{M} = \mathsf{M}; \langle \mathtt{x}\rangle \overset{\mathsf{M}}{\mapsto} 5\right\}$, at which point the Write rule can be applied. Note also that Alloc does not require specific permissions on the block in the postcondition; if they turn out to be RO, the resulting goal is unsolvable.

Unsurprisingly, the rule for accessing a memory cell just for reading purposes requires no adjustments since any permission allows reading. Moreover, the Call rule for method invocation does not need adjustments either. Below, we describe how borrow and return seamlessly operate within a method call:

$$\textsc{Call}\quad \frac{\begin{array}{c}\mathcal{F} \triangleq \mathtt{f}(\overline{\mathtt{x_i}}) : \{\phi_{\mathtt{f}}; \mathtt{P_f}\}\{\psi_{\mathtt{f}}; \mathtt{Q_f}\} \in \Sigma \qquad \mathtt{R} = [\sigma]\mathtt{P_f} \qquad \vdash \phi \Rightarrow [\sigma]\phi_{\mathtt{f}} \quad \overline{\mathtt{e_i}} = [\sigma]\overline{\mathtt{x_i}} \\ \mathsf{Vars}(\overline{\mathtt{e_i}}) \subseteq \Gamma \quad \phi' \triangleq [\sigma]\psi_{\mathtt{f}} \qquad \mathtt{R}' \triangleq [\sigma]\mathtt{Q_f} \quad \Sigma; \Gamma; \{\phi \wedge \phi'; \mathtt{P} * \mathtt{R}'\} \rightsquigarrow \{\mathcal{Q}\} \big| \, \mathtt{c}\end{array}}{\Sigma; \Gamma; \{\phi; \mathtt{P} * \mathtt{R}\} \rightsquigarrow \{\mathcal{Q}\} \big| \, \mathtt{f}(\overline{\mathtt{e_i}}); \mathtt{c}}$$

The Call rule fires when a sub-heap $\mathtt{R}$ in the precondition of the goal can be unified with the precondition $\mathtt{P_f}$ of a function $\mathtt{f}$ from context $\Sigma$. Some salient points are worth mentioning here: (1) the *annotation borrowing* from $\mathtt{R}$ to $\mathtt{P_f}$ for those symbolic sub-heaps in $\mathtt{P_f}$ which require read-only permissions is handled by the unification of $\mathtt{P_f}$ with $\mathtt{R}$, namely $\mathtt{R} = [\sigma]\mathtt{P_f}$ (*i.e.*, substitution accounts for borrows: $\alpha/\mathsf{a}$); (2) the *annotation recovery* in the new precondition is implicit via $\mathtt{R}' \triangleq [\sigma]\mathtt{Q}_\mathtt{f}$, where the substitution $\sigma$ was computed during the unification, that is, while borrowing; (3) finding a substitution $\sigma$ for $\mathtt{R} = [\sigma]\mathtt{P}_\mathtt{f}$ fails if $\mathtt{R}$ does not have sufficient accessibility permissions to call $\mathtt{f}$ (*i.e.*, substitutions of the form $\mathsf{a}/\mathsf{M}$ are disallowed since the domain of $\sigma$ may only contain existentials). We reiterate that read-only specifications only manipulate symbolic borrows, that is to say, $\mathsf{RO}$ constants are not expected in the specification.

#### 3.2 Memory Model

We closely follow the standard SL memory model [32,37] and assume Loc ⊂ Val.

(Heap) $\mathtt{h} \in \text{Heaps} ::= \text{Loc} \rightharpoonup \text{Val}$ (Stack) $\mathtt{s} \in \text{Stacks} ::= \text{Var} \rightarrow \text{Val}$

To enable C-like accounting of dynamically-allocated memory blocks, we assume that the heap $\mathtt{h}$ also stores sizes of allocated blocks in dedicated locations. Conceptually, this part of the heap corresponds to the meta-data of the memory allocator. This accounting ensures that only a previously allocated memory block can be disposed (as opposed to any set of allocated locations), enabling the `free` command to accept a single argument, the address of the block. To model this meta-data, we introduce a function $\mathtt{bl}\colon \text{Loc} \to \text{Loc}$, where $\mathtt{bl}(\mathtt{x})$ denotes the location in the heap where the block meta-data for the address $\mathtt{x}$ is stored, if $\mathtt{x}$ is the starting address of a block. In an actual language implementation, $\mathtt{bl}(\mathtt{x})$ might be, *e.g.*, $\mathtt{x} - \mathtt{1}$ (*i.e.*, the meta-data is stored right before the block).

Since we have opted for an unsophisticated permission mechanism, where the *heap ownership is not divisible*, but some heap locations are restricted to $\mathsf{RO}$, the definition of the satisfaction relation $\models_{\mathcal{I}}^{\Sigma,\mathtt{R}}$ for the annotated assertions in a particular context $\Sigma$ and given an interpretation $\mathcal{I}$, is parameterised with a fixed set of read-only locations, $\mathtt{R}$:

- $\langle \mathtt{h}, \mathtt{s}\rangle \models_{\mathcal{I}}^{\Sigma,\mathtt{R}} \{\phi; \mathsf{emp}\}$ *iff* $[\![\phi]\!]_\mathtt{s} = \mathsf{true}$ and $\mathsf{dom}\,(\mathtt{h}) = \emptyset$.
- $\langle \mathtt{h}, \mathtt{s}\rangle \models_{\mathcal{I}}^{\Sigma,\mathtt{R}} \{\phi; \langle \mathsf{e}_1, \iota\rangle \overset{\alpha}{\mapsto} \mathsf{e}_2\}$ *iff* $[\![\phi]\!]_\mathtt{s} = \mathsf{true}$ and $\mathtt{l} \triangleq [\![\mathsf{e}_1]\!]_\mathtt{s} + \iota$ and $\mathsf{dom}\,(\mathtt{h}) = \{\mathtt{l}\}$ and $\mathtt{h}(\mathtt{l}) = [\![\mathsf{e}_2]\!]_\mathtt{s}$ and $\mathtt{l} \in \mathtt{R} \Leftrightarrow \alpha = \mathsf{RO}$ .
- $\langle \mathtt{h}, \mathtt{s}\rangle \models_{\mathcal{I}}^{\Sigma,\mathtt{R}} \{\phi; [\mathsf{e}, n]^\alpha\}$ *iff* $[\![\phi]\!]_\mathtt{s} = \mathsf{true}$ and $\mathtt{l} \triangleq \mathtt{bl}([\![\mathsf{e}]\!]_\mathtt{s})$ and $\mathsf{dom}\,(\mathtt{h}) = \{\mathtt{l}\}$ and $\mathtt{h}(\mathtt{l}) = n$ and $\mathtt{l} \in \mathtt{R} \Leftrightarrow \alpha = \mathsf{RO}$.
- $\langle \mathtt{h}, \mathtt{s}\rangle \models_{\mathcal{I}}^{\Sigma,\mathtt{R}} \{\phi; \mathtt{P}_1 * \mathtt{P}_2\}$ *iff* $\exists\, \mathtt{h}_1, \mathtt{h}_2, \mathtt{h} = \mathtt{h}_1 \uplus \mathtt{h}_2$ and $\langle \mathtt{h}_1, \mathtt{s}\rangle \models_{\mathcal{I}}^{\Sigma,\mathtt{R}} \{\phi; \mathtt{P}_1\}$ and $\langle \mathtt{h}_2, \mathtt{s}\rangle \models_{\mathcal{I}}^{\Sigma,\mathtt{R}} \{\phi; \mathtt{P}_2\}$.
- $\langle \mathtt{h}, \mathtt{s}\rangle \models_{\mathcal{I}}^{\Sigma,\mathtt{R}} \{\phi; \mathsf{p}(\overline{\psi_\mathtt{i}})\}$ *iff* $[\![\phi]\!]_\mathtt{s} = \mathsf{true}$ and $\mathcal{D} \triangleq \mathsf{p}(\overline{\mathtt{x}_\mathtt{i}})\overline{\langle \mathsf{e}_\mathtt{k}, \{\chi_\mathtt{k}, \mathtt{R}_\mathtt{k}\}\rangle} \in \Sigma$ and $\left\langle \mathtt{h}, \overline{[\![\psi_\mathtt{i}]\!]_\mathtt{s}} \right\rangle \in \mathcal{I}(\mathcal{D})$ and $\bigvee_\mathtt{k}(\langle \mathtt{h}, \mathtt{s}\rangle \models_{\mathcal{I}}^{\Sigma,\mathtt{R}} [\overline{\psi_\mathtt{i}/\mathtt{x}_\mathtt{i}}]\{\phi \wedge \mathsf{e}_\mathtt{k} \wedge \chi_\mathtt{k}; \mathtt{R}_\mathtt{k}\})$.

There are two non-standard cases: points-to and block, whose permissions must agree with $\mathtt{R}$. Note that in the definition of satisfaction, we only need to consider that case where the permission $\alpha$ is a value (*i.e.*, either $\mathsf{RO}$ or $\mathsf{M}$). Although in a specification $\alpha$ can also be a variable, well-formedness guarantees that this variable must be logical, and hence will be substituted away in the definition of validity. We stress the fact that a reference that has $\mathsf{RO}$ permissions to a certain symbolic heap still retains the full ownership of that heap, with the restriction that it is not allowed to update or deallocate it. Note that deallocation additionally requires a mutable permission for the enclosing block.

#### 3.3 Soundness

The BoSSL operational semantics is in the spirit of the traditional SL [38], and hence is omitted for the sake of saving space (selected rules are available in the extended version of the paper). The validity definition and the soundness proofs of SSL are ported to BoSSL without any modifications, since our current definition of satisfaction implies the one defined for SSL:

**Definition 1 (Validity).** *We say that a well-formed Hoare-style specification* $\Sigma; \Gamma; \{\mathcal{P}\}\ \mathsf{c}\ \{\mathcal{Q}\}$ *is* valid wrt. *the function dictionary* $\Delta$ iff *whenever* $\mathsf{dom}\,(\mathtt{s}) = \Gamma$*,* $\forall \sigma_{gv} = \overline{[\mathtt{x_i} \mapsto \mathtt{d_i}]}_{\mathtt{x_i} \in \mathsf{GV}(\Gamma, \mathcal{P}, \mathcal{Q})}$ *such that* $\langle \mathtt{h}, \mathtt{s} \rangle \models^{\Sigma}_{\mathcal{I}} [\sigma_{gv}]\mathcal{P}$*, and* $\Delta; \langle \mathtt{h}, (\mathtt{c}, \mathtt{s}) \cdot \epsilon \rangle \rightsquigarrow^{*} \langle \mathtt{h}', (\mathbf{skip}, \mathtt{s}') \cdot \epsilon \rangle$*, it is also the case that* $\langle \mathtt{h}', \mathtt{s}' \rangle \models^{\Sigma}_{\mathcal{I}} [\sigma_{ev} \uplus \sigma_{gv}]\mathcal{Q}$ *for some* $\sigma_{ev} = \overline{[\mathtt{y_j} \mapsto \mathtt{d_j}]}_{\mathtt{y_j} \in \mathsf{EV}(\Gamma, \mathcal{P}, \mathcal{Q})}$.

The following theorem guarantees that, given a program c generated with BoSSL, a heap model, and a set of read-only locations R that satisfy the program's precondition, executing c does not change those read-only locations:

**Theorem 1 (RO Heaps Do Not Change).** *Given a Hoare-style specification* $\Sigma; \Gamma; \{\phi; \mathtt{P}\}\mathtt{c}\{\mathcal{Q}\}$*, which is valid* wrt. *the function dictionary* $\Delta$*, and a set of read-only memory locations* R*, if:*

(i) $\langle \mathtt{h}, \mathtt{s} \rangle \models^{\Sigma, \mathtt{R}}_{\mathcal{I}} [\sigma]\mathcal{P}$*, for some* $\mathtt{h}, \mathtt{s}$ *and* $\sigma$*, and*
(ii) $\Delta; \langle \mathtt{h}, (\mathtt{c}, \mathtt{s}) \cdot \epsilon \rangle \rightsquigarrow^{*} \langle \mathtt{h}', (\mathtt{c}', \mathtt{s}') \cdot \epsilon \rangle$ *for some* $\mathtt{h}', \mathtt{s}'$ *and* $\mathtt{c}'$
(iii) $\mathtt{R} \subseteq \mathsf{dom}\,(\mathtt{h})$

*then* $\mathtt{R} \subseteq \mathsf{dom}\,(\mathtt{h}')$ *and* $\forall \mathtt{l} \in \mathtt{R},\ \mathtt{h}(\mathtt{l}) = \mathtt{h}'(\mathtt{l})$.

Starting from an abstract state where a spatial heap has a read-only permission, under no circumstance can this permission be strengthened to M:

**Corollary 1 (No Permission Strengthening).** *Given a valid Hoare-style specification* $\Sigma; \Gamma; \{\phi; \mathtt{P}\}\ \mathtt{c}\ \{\psi; \mathtt{Q}\}$ *and a permission* $\alpha$*, if* $\psi \Rightarrow (\alpha = \mathsf{M})$ *then it is also the case that* $\phi \Rightarrow (\alpha = \mathsf{M})$ .

As it turns out, permission weakening is possible, since, though problematic, postcondition weakening is sound in general. However, even though this affects completeness, it does not affect our termination results. For example, given a synthesised auxiliary function $\mathcal{F} \triangleq \mathtt{f}(\mathtt{x}, \mathtt{r}) : \left\{\mathtt{x} \overset{\mathsf{a_1}}{\mapsto} \mathtt{t} * \mathtt{r} \overset{\mathsf{M}}{\mapsto} \mathtt{x}\right\}\left\{\mathtt{x} \overset{\mathsf{a_2}}{\mapsto} \mathtt{t} * \mathtt{r} \overset{\mathsf{M}}{\mapsto} \mathtt{t} + 1\right\}$, and a synthesis goal $\Sigma, \mathcal{F}; \Gamma; \left\{\mathtt{x} \overset{\mathsf{M}}{\mapsto} 7 * \mathtt{y} \overset{\mathsf{M}}{\mapsto} \mathtt{x}\right\} \rightsquigarrow \left\{\mathtt{x} \overset{\mathsf{M}}{\mapsto} 7 * \mathtt{y} \overset{\mathsf{M}}{\mapsto} \mathtt{z}\right\} \Big| \mathtt{c}$, firing the Call rule for the candidate function $\mathtt{f}(\mathtt{x}, \mathtt{r})$ would lead to the unsolvable goal $\Sigma, \mathcal{F}; \Gamma;$ $\left\{\mathtt{x} \overset{\mathsf{a_2'}}{\mapsto} 7 * \mathtt{y} \overset{\mathsf{M}}{\mapsto} 8\right\} \rightsquigarrow \left\{\mathtt{x} \overset{\mathsf{M}}{\mapsto} 7 * \mathtt{y} \overset{\mathsf{M}}{\mapsto} \mathtt{z}\right\} \Big| \mathtt{f}(\mathtt{x}, \mathtt{y}); \mathtt{c}$. Frame may never be fired on this new goal since the permission of reference x in the goal's precondition has been permanently weakened. To eliminate such sources of incompleteness we require the user-provided predicates and specifications to be well-formed:

**Definition 2 (Well-Formedness of Spatial Predicates).** *We say that a spatial predicate* $\mathsf{p}(\overline{\mathtt{x_i}})\ \overline{\langle \mathsf{e_k}, \{\chi_\mathsf{k}, \mathsf{R_k}\} \rangle}_{\mathtt{k} \in 1..\mathtt{N}}$ *is* well-formed iff

$$\left(\bigcup_{\mathtt{k}=1}^{\mathtt{N}} (\mathsf{Vars}\,(\mathsf{e_k}) \cup \mathsf{Vars}\,(\chi_\mathsf{k}) \cup \mathsf{Vars}\,(\mathtt{R_k})) \cap \mathtt{Perm}\right) \subseteq (\overline{\mathtt{x_i}} \cap \mathtt{Perm}).$$

That is, every accessibility annotation within the predicate's clause is bound by the predicate's parameters.

**Definition 3 (Well-Formedness of Specifications).** *We say that a Hoare-style specification* $\Sigma; \Gamma; \{\mathcal{P}\}\ c\ \{\mathcal{Q}\}$ *is* well-formed iff $\mathsf{EV}(\Gamma, \mathcal{P}, \mathcal{Q}) \cap \mathtt{Perm} = \emptyset$ *and every predicate instance in* $\mathcal{P}$ *and* $\mathcal{Q}$ *is an instance of a well-formed predicate.*

That is, postconditions are not allowed to have existential accessibility annotations in order to avoid permanent weakening of accessibility.

A callee that requires borrows for a symbolic heap always returns back to the caller its original permission for that respective symbolic heap:

**Corollary 2 (Borrows Always Return).** *A heaplet with permission* $\alpha$*, either (a) retains the same permission* $\alpha$ *after a call to a function that is decorated with well-formed specifications and that requires for that heaplet to have read-only permission, or (b) it may be deallocated in case if* $\alpha = \mathsf{M}$.

## 4 Implementation and Evaluation

We implemented BoSSL in an enhanced version of the SuSLik tool, which we refer to as ROBoSuSLik [12].[11] The changes to the original SuSLik infrastructure affected less than 100 lines of code. The extended synthesis is backwards-compatible with the original benchmarks. To make this possible, we treat the original SSL specifications as annotated/instantiated with $\mathsf{M}$ permissions, whenever necessary, which is consistent with treatment of access permissions in BoSSL.

We have conducted an extensive experimental evaluation of ROBoSuSLik, aiming to answer the following research questions:

1. Do borrowing annotations improve the *performance* of SSL-based synthesis when using standard search strategy [34, § 5.2]?
2. Do read-only borrows improve the *quality* of synthesised programs, in terms of size and comprehensibility, *wrt.* to their counterparts obtained from regular, "all-mutable" specifications?
3. Do we obtain *stronger correctness guarantees* for the programs from the standard SSL benchmark suite [34, § 6.1] by simply adding, whenever reasonable, read-only annotations to their specifications?
4. Do borrowing specifications enable more *robust* synthesis? That is, should we expect to obtain better programs/synthesis performance on average *regardless* of the adopted unification and search strategies?

### 4.1 Experimental Setup

*Benchmark Suite.* To tackle the above research questions, we have adopted most of the heap-manipulating benchmarks from SuSLik suite [34, § 6.1] (with some variations) into our sets of experiments. In particular we looked at the group of benchmarks which manipulate singly linked list segments, sorted linked list segments and binary trees. We did not include the benchmarks concerning binary search trees (BSTs) for the reasons outlined in the next paragraph.

[11] The sources are available at `https://github.com/TyGuS/robosuslik`.

*The Tools.* For a fair comparison which accounts for the latest advancements to SuSLik, we chose to parameterise the synthesis process with a flag that turns the read-only annotations on and off (off means that they are set to be mutable). Those values which are the result of having this flag set will be marked in the experiments with RO, while those marked with Mut ignore the read-only annotations during the synthesis process. For simplicity, we will refer to the two instances of the tool, namely RO and Mut, as two different tools. Each tool was set to timeout after 2 minutes of attempting to synthesise a program.

*Criteria.* In an attempt to quantify our results, we have looked at the size of the synthesised program (*AST size*), the absolute time needed to synthesise the code given its specification, averaged over several runs (*Time*), the number of backtrackings in the proof search due to nondeterminism (*#Backtr*), the total number of rule applications that the synthesis fired during the search (*#Rules*), including those that lead to unsolvable goals, and the strength of the guarantees offered by the specifications (*Stronger Guarantees*).

*Variables.* Some benchmarks have shown improvement over the synthesis process without the read-only annotations. To emphasise the fact that read-only annotations' improvements are not accidental, we have varied the inductive definitions of the corresponding benchmarks to experiment with different properties of the underlying structure: the shape of the structure (in all the definitions), the length of the structure (for those benchmarks tagged with *len*), the values stored within the structure (*val*), a combination of all these properties (*all*) as well as with the sortedness property for the "Sorted list" group of benchmarks.

*Experiment Schema.* To measure the performance and the quality of the borrowing-aware synthesis we ran the benchmarks against the two different tools and did a one-to-one comparison of the results. We ran each tool three times for each benchmark, and average the resulted synthesis time. All the other evaluation criteria remain constant within all three runs.

To measure the tools' robustness we stressed the synthesis algorithm by altering the default proof search strategy. We prepared 42 such perturbations which we used to run against the different program variants enumerated above. Each pair of program variant and proof strategy perturbation has been then analysed to measure the number of rules that had been fired by RO and Mut.

*Hardware Setup.* The experiments were conducted on a 64-bit machine running Ubuntu, with an Intel Xeon CPU (6 cores, 2.40GHz) with 32GB RAM.

#### 4.2 Performance and Quality of the Borrowing-Aware Synthesis

Tab. 1 captures the results of running RO and Mut against the considered benchmarks. It provides the empirical proof that the borrowing-aware synthesis improves the performance of the original SSL-based synthesis, or in other words, answering positively the Research Question 1. RO suffers almost no loss in performance (except for a few cases, such as the list segment `append` where there is a negligible increase in time), while the gain is considerable for those synthesis problems with complex pointer manipulation. For example, if we consider the number of fired rules as the performance measurement criteria, in the worst

| Group | Description | AST size | | Time (sec) | | | #Backtr. | | | #Rules | | | Stronger Guarant. |
|---|---|---|---|---|---|---|---|---|---|---|---|---|---|
| | | RO | Mut | RO | Mut | Mut/RO | RO | Mut | Mut/RO | RO | Mut | Mut/RO | |
| Linked List Segment | append | 20 | 20 | 1.5 | 1.4 | 0.9x | 8 | 8 | 1.0x | 77 | 78 | 1.0x | YES |
| | delete | 44 | 44 | 1.9 | 2.1 | 1.1x | 67 | 67 | 1.0x | 180 | 180 | 1.0x | same |
| | dispose | 11 | 11 | 0.5 | 0.5 | 1.0x | 0 | 0 | 1.0x | 8 | 8 | 1.0x | same |
| | init | 13 | 13 | 0.7 | 0.7 | 1.0x | 5 | 5 | 1.0x | 27 | 27 | 1.0x | YES |
| | lcopy | 32 | 35 | 1.0 | 1.0 | 1.0x | 9 | 14 | 1.5x | 66 | 82 | 1.2x | YES |
| | length | 22 | 22 | 1.5 | 1.5 | 1.0x | 2 | 2 | 1.0x | 38 | 38 | 1.0x | YES |
| | max | 28 | 28 | 1.4 | 1.5 | 1.1x | 2 | 2 | 1.0x | 38 | 38 | 1.0x | YES |
| | min | 28 | 28 | 1.5 | 1.5 | 1.0x | 2 | 2 | 1.0x | 38 | 38 | 1.0x | YES |
| | singleton | 11 | 11 | 0.5 | 0.5 | 1.0x | 8 | 8 | 1.0x | 30 | 30 | 1.0x | same |
| Sorted List | ins-sort-all | 29 | 29 | 3.7 | 3.8 | 1.0x | 5 | 5 | 1.0x | 60 | 60 | 1.0x | YES |
| | ins-sort-len | 29 | 29 | 3.0 | 3.0 | 1.0x | 7 | 8 | 1.1x | 59 | 60 | 1.0x | YES |
| | ins-sort-val | 29 | 29 | 2.6 | 2.5 | 1.0x | 5 | 5 | 1.0x | 57 | 57 | 1.0x | YES |
| | insert | 53 | 53 | 7.8 | 8.0 | 1.0x | 35 | 96 | 2.7x | 214 | 338 | 1.6x | YES |
| | prepend | 11 | 11 | 0.5 | 0.6 | 1.2x | 1 | 1 | 1.0x | 17 | 17 | 1.0x | YES |
| Tree | dispose | 16 | 16 | 0.4 | 0.5 | 1.2x | 0 | 0 | 1.0x | 10 | 10 | 1.0x | same |
| | flatten-acc | 35 | 35 | 2.1 | 2.0 | 1.0x | 24 | 24 | 1.0x | 118 | 118 | 1.0x | same |
| | flatten-app | 48 | 48 | 1.6 | 1.7 | 1.0x | 14 | 14 | 1.0x | 76 | 76 | 1.0x | same |
| | morph | 19 | 19 | 0.6 | 0.5 | 1.0x | 1 | 1 | 1.0x | 24 | 24 | 1.0x | YES |
| | tcopy-all | 42 | 51 | 1.5 | 2.2 | 1.5x | 10 | 88 | 8.8x | 85 | 296 | 3.5x | YES |
| | tcopy-len | 36 | 42 | 1.3 | 2.0 | 1.5x | 6 | 90 | 15x | 72 | 304 | 4.2x | YES |
| | tcopy-val | 42 | 51 | 1.4 | 5.3 | 3.8x | 10 | 1222 | 122x | 82 | 2673 | 32x | YES |
| | tcopy-ptr-all | 46 | 55 | 1.6 | 2.4 | 1.5x | 10 | 88 | 8.8x | 93 | 303 | 3.3x | YES |
| | tcopy-ptr-len | 40 | 46 | 1.3 | 2.2 | 1.7x | 6 | 90 | 15x | 80 | 311 | 3.9x | YES |
| | tcopy-ptr-val | 46 | 55 | 1.3 | 5.8 | 4.5x | 10 | 1222 | 122x | 89 | 2679 | 30x | YES |
| | tsize-all | 32 | 38 | 1.5 | 1.4 | 0.9x | 2 | 4 | 2.0x | 45 | 51 | 1.1x | YES |
| | tsize-len | 32 | 32 | 1.2 | 1.1 | 0.9x | 2 | 2 | 1.0x | 44 | 46 | 1.0x | YES |
| | tsize-ptr-all | 36 | 42 | 1.6 | 1.4 | 0.9x | 2 | 4 | 2.0x | 53 | 58 | 1.1x | YES |
| | tsize-ptr-len | 36 | 36 | 1.3 | 1.3 | 1.0x | 2 | 2 | 1.0x | 52 | 53 | 1.0x | YES |

Table 1: Benchmarks and comparison between the results for synthesis with read-only annotations (RO) and without them (Mut). For each case study we measure the *AST size* of the synthesised program, the *Time* needed to synthesize the benchmark, the number of times that the synthesiser had to discard a derivation branch (*#Backtr.*), and the total number of fired rules (*#Rules*).

case, RO behaves the same as Mut, while in the best scenario it buys us a 32-fold decrease in the number of applied rules. At the same time, synthesising a few small examples in the RO case is a bit slower, despite the same or smaller number of rule applications. This is due to the increased number of logical variables (because of added borrows) when discharging obligations via SMT solver.

Fig. 5 offers a statistical view of the numbers in the table, where smaller bars mark a better performance. The barplots indicate that as the complexity of the problem increases (approximately from left to right), RO outperforms Mut.

Perhaps the most important take-away from this experiment is that the synthesis with read-only borrows often produces a more concise program (light green cells in the columnt *AST size* of Tab. 1), while retaining the same or better performance *wrt.* all the evaluated criteria. For instance, RO gets rid of the spurious write from the motivating example introduced in Sec. 1, reducing the AST size from 35 nodes down to 32, while in the same time firing fewer rules. That also means that we secure a positive answer for Research Question 2.

### 4.3 Stronger Correctness Guarantees

To answer Research Question 3, we have manually compared the guarantees offered by the specifications annotated with RO permissions against the default

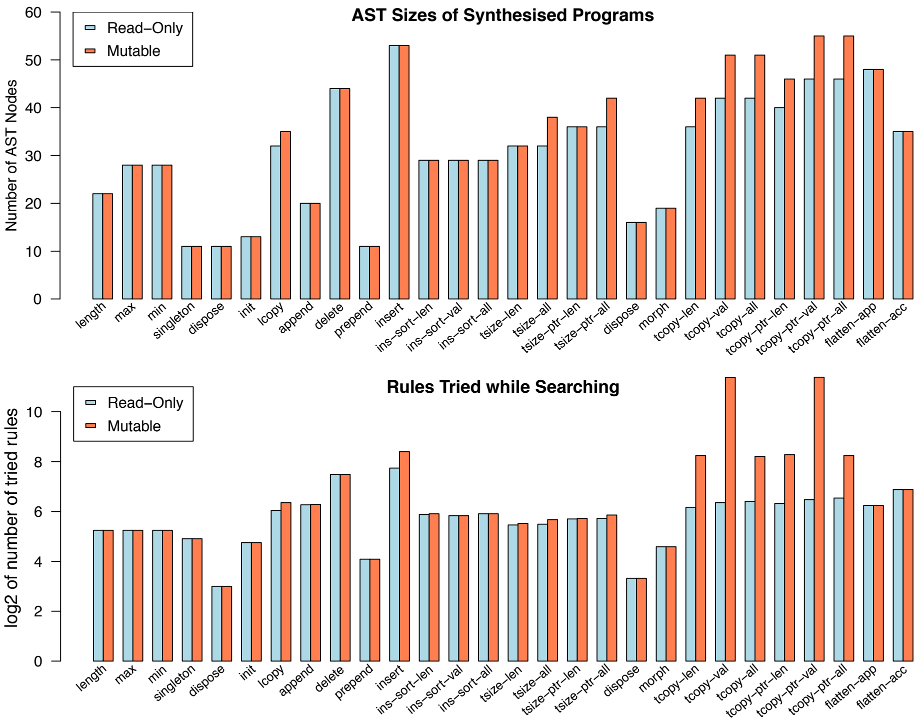

Fig. 5: Statistics for synthesis with and without Read-Only specifications.

ones - the results are summarized in the last column of Tab. 1. For instance, a specification stating that the shape of a linked-list segment is read-only implies that the size of that segment remains constant through the program's execution. In other words, the length property need not be captured separately in the segment's definition. If, in addition to the shape, the payload of the segment is also read-only, then the set of values and their ordering are also invariant.

Consider the goal $\{\mathsf{lseg}(x, y, s, a_1, a_2, a_3)\} \leadsto \{\mathsf{lseg}(x, y, s, a_1, a_2, a_3)\}$, where lseg is an inductive definition of a list segment which ends at `y` and contains the set of values `s`. The borrowing-aware synthesiser will produce a program which is guaranteed to treat the segment pointed by `x` and ending with `y` as read-only (that is, its shape, length, values and orderings are invariant). At the same time, for a goal $\{\mathsf{lseg}(x, y, s)\} \leadsto \{\mathsf{lseg}(x, y, s)\}$ , the guarantees are that the returned segment still ends in `y` and contains values `s`. Internal modifications of the segment, such as reordering and duplicating list elements, may still occur.

The few entries marked with `same` are programs with specifications which have not got stronger when instrumented with RO annotations (*e.g.*, `delete`). These benchmarks require mutation over the entire data structure, hence the read-only annotations do not influence the offered guarantees. Overall, our observations that read-only annotations offer stronger guarantees are in agreement with the works on SL-based program verification [9, 13], but are promoted here to the more challenging problem of program synthesis.

#### 4.4 Robustness under Synthesis Perturbations

There is no single search heuristics that will work equally well for any given specification: for a particular fixed search strategy, a synthesiser can exhibit suboptimal performance for some goals, while converging quickly on some others. By evaluating robustness *wrt.* to RO and M specification methodologies, we are hoping to show that, provided a large variety of "reasonable" search heuristics, read-only annotations deliver better synthesis performance "on average".

For this set of experiments, we have focused on four characteristic programs from our performance benchmarks based on their pointer manipulation complexity: list segment copy (`lcopy`), insertion into a sorted list segment (`insert`), copying a tree (`tcopy`), and a variation of the tree copy that shares the same pointer for the input tree and its returned copy (`tcopy-ptr`).

*Exploring Different Unification Orders.* Since spatial unification stays at the core of the synthesis process, we implemented 6 different strategies for choosing a unification candidate based on the following criteria: the size of the heaplet chunk (favor the smallest heap vs. the largest one as the best unification candidate), the name of the predicate (we considered both an ascending as well as a descending priority queue), and a customised ranking function which associates a cost to a symbolic heap based on its kind—a block is cheaper to unify than a points-to which in turn is cheaper than a spatial predicate.

*Exploring Different Search Strategies.* We next designed 6 strategies for prioritising the rule applications. One of the crux rules in this matter, is the Write rule whose different priority schemes might make all the results seem randomly-generated. In the cases where Write leads to unsolvable goals, one might rightfully argue that RO has a clear advantage over Mut (*fail fast*). However, for the cases where mutation leads to a solution faster, then Mut might have an advantage over RO (*solve fast*). Because these are just intuitive observations, and for fairness sake, we experimented with both the cases where Write has a high and a low priority in the queue of rule phases [34, § 5.2]. Since most of the benchmarks involve recursion, we also chose to shuffle around the priorities of the Open and Call rules. Again, we chose between a stack high and a bottom low priority for these rules to give a fair chance to both tools.

We considered all combinations of the 6 unification permutations and the 6 rule-application permutations (plus the default one) to obtain 42 different proof search perturbations. We will use the following notation in the narrative below:

– `S` is the set comprising the synthesis problems: `lcopy`, `insert`, `tcopy`, `tcopy-ptr`.
– `V` is the set of all specification variations: *len*, *val*, *all*.
– `K` is the set of all 42 possible tool perturbations.

The distributions of the number of rules fired for each tool (RO and Mut) with the 42 perturbations over the 4 synthesis problems with 3 variants of specification each, that is 1008 different synthesis runs, are summarised using the boxplots in Fig. 6. There is a boxplot corresponding to each pair of tool and synthesis problem. In the ideal case, each boxplot contains 126 data points corresponding to a unique combination $(\mathtt{v}, \mathtt{k})$ of a specification variation $\mathtt{v} \in \mathtt{V}$ and a tool perturbation $\mathtt{k} \in \mathtt{K}$. A boxplot is the distribution of such data based on a

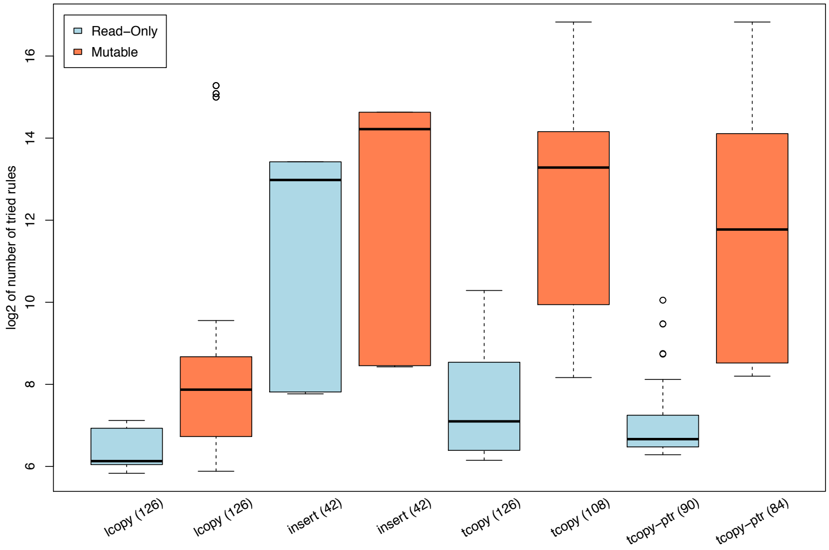

Fig. 6: Boxplots of variations in $\log_2$(numbers of applied rules) for synthesis perturbations. Numbers of data points for each example are given in parentheses.

six number summary: minimum, first quartile, median, third quartile, maximum, outliers. For example, the boxplot for `tcopy-ptr` corresponding to RO and containing 90 data points, reads as follows: "the synthesis processes fired between 64 and 256 rules, with most of the processes firing between 64 and 128 rules. There are three exception where the synthesiser fired more than 256 rules". Note that the y-axis represents the binary logarithm of the number of fired rules.

Even though we attempted to synthesise each program 126 times for each tool, some attempts hit the timeout and therefore their corresponding data points had to be eliminated from the boxplot. It is of note, though, that whenever RO with configuration $(\mathtt{v}, \mathtt{k})$ hit the timeout for the synthesis problem $\mathtt{s} \in \mathtt{S}$, so did Mut, hence both the $(\mathsf{RO}, \mathtt{s}, (\mathtt{v}, \mathtt{k}))$ as well as $(\mathsf{Mut}, \mathtt{s}, (\mathtt{v}, \mathtt{k}))$ are omitted from the boxplots. But the inverse did not hold: RO hit the timeout fewer times than Mut, hence RO is measured at disadvantage (*i.e.*, more data points means more opportunities to show worse results). Since `insert` collected the highest number of timeouts, we equalised it to remove non-matched entries across the two tools.

Despite RO's potential measurement disadvantage, the boxplots depicts it as a clear winner. Not only RO fires fewer rules in all the cases, but with the exception of `insert`, it is also more stable to the proof search perturbations, it varies a few order of magnitude less than Mut does for the same configurations. Fig. 7 supports this observation by offering a more detailed view on the distributions of the numbers of fired rules per synthesis configuration. Taller bars show that more processes fall in the same range (*wrt.* the number of fired rules). For `lcopy`, `tcopy`, `tcopy-ptr` it is clear that Mut has a wider distribution of the number of fired rules, that is, Mut is *more sensitive* to the perturbations than RO. We additionally make some further observations:

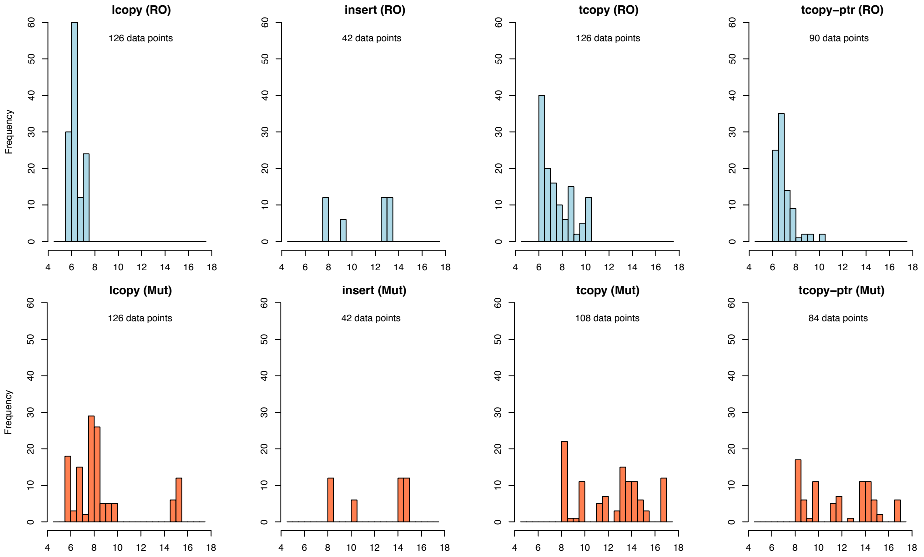

Fig. 7: Distributions of $\log_2$(number of attempted rule applications).

– Despite a similar distribution *wrt.* the numbers of fired rules in the case of `insert`, RO produces compact ASTs of size 53 for all perturbations, while Mut fluctuates between producing ASTs of size 53 and 62.
– For all the synthesis tasks, RO produced the same AST irrespective of the tool's perturbation. In contrast, there were synthesis problems for which Mut produced as many as 3 different ASTs for different perturbations, none of which were as concise as the one produced by RO for the same configuration.
– The outliers of (Mut, `lcopy`) are ridiculously high, firing close to 40k rules.
– The outliers of (RO, `tcopy`) are still below the median values of (Mut, `tcopy`).
– Except for `insert`, the best performance of Mut, in terms of fired rules, barely overlaps with the worst performance of RO.
– Except for `insert`, the medians of RO are closer to the lowest value of the data distribution, as opposed to Mut where the tendancy is to fire more rules.
– In absolute values, RO hit the 2-minutes timeout 102 times compared to Mut, which hit the timeout 132 times.

We believe that the main take-aways from this set of experiments, along with the positive answer to the Research Question 4, are as follows:
– RO is more stable *wrt.* the number of rules fired and the size of the generated AST for many reasonable proof search perturbations.
– RO produces better programs, which avoid spurious statements, irrespective of the perturbation and number of rules fired during the search.

### 5 Limitations and Discussion

*Flexible aliasing.* Separating conjunction asserts that the heap can be split into two disjoint parts, or in other words it carries an implicit non-aliasing information. Specifically, $\mathtt{x} \mapsto \_ * \mathtt{y} \mapsto \_$ states that `x` and `y` are non-aliased. Such assertions can be used to specify methods as below:

$$\{\mathtt{x} \mapsto \mathtt{n} * \mathtt{y} \mapsto \mathtt{m} * \mathtt{ret} \mapsto \mathtt{x}\}\ \mathtt{sum(x,\ y,\ ret)}\ \{\mathtt{x} \mapsto \mathtt{n} * \mathtt{y} \mapsto \mathtt{m} * \mathtt{ret} \mapsto \mathtt{n} + \mathtt{m}\}$$

Occasionally, enforcing `x` and `y` to be non-aliased is too restrictive, rejecting safe calls such as $\mathtt{sum}(\mathtt{p}, \mathtt{p}, \mathtt{q})$. Approaches to support immutable annotations permit such calls without compromising safety if both pointers, aliased or not, are annotated as read-only [9,13]. BoSSL does not support such flexible aliasing.

*Precondition strengthening.* Let us assume that $\mathsf{srtl}(\mathtt{x}, \mathtt{n}, \mathtt{lo}, \mathtt{hi}, \alpha_1, \alpha_2, \alpha_3)$ is an inductive predicate that describes a sorted linked list of size `n` with `lo` and `hi` being the list's minimum and maximum payload value, respectively. Now, consider the following synthesis goal:

$$\{\mathtt{x}, \mathtt{y}\}\,; \{\mathtt{y} \mapsto \mathtt{x} * \mathsf{srtl}(\mathtt{x}, \mathtt{n}, \mathtt{lo}, \mathtt{hi}, \mathsf{M}, \mathsf{M}, \mathsf{M})\} \rightsquigarrow \{\mathtt{y} \mapsto \mathtt{n} * \mathsf{srtl}(\mathtt{x}, \mathtt{n}, \mathtt{lo}, \mathtt{hi}, \mathsf{M}, \mathsf{M}, \mathsf{M})\}.$$

As stated, the goal clearly requires the program to compute the length `n` of the list. Imagine that we already have a function that does precisely that, even though it is stated in terms of a list predicate that does not enforce sortedness:

$$\{\mathtt{ret} \mapsto \mathtt{x} * \mathsf{ls}(\mathtt{x}, \mathtt{n}, \mathsf{a}_1, \mathsf{a}_2, \mathsf{a}_3)\}\ \mathtt{length(x,\ ret)}\ \{\mathtt{ret} \mapsto \mathtt{n} * \mathsf{ls}(\mathtt{x}, \mathtt{n}, \mathsf{a}_1, \mathsf{a}_2, \mathsf{a}_3)\}$$

To solve the initial goal, the synthesiser could weaken the given precondition $\mathsf{srtl}(\mathtt{x}, \mathtt{n}, \mathtt{lo}, \mathtt{hi}, \mathsf{M}, \mathsf{M}, \mathsf{M})$ to $\mathsf{ls}(\mathtt{x}, \mathtt{n}, \mathsf{M}, \mathsf{M}, \mathsf{M})$, and then successfully synthesise a call to the `length` method. Unfortunately, the resulting goal, obtained after having emitted the call to `length` and applying Frame, is unsolvable:

$$\{\mathtt{x}, \mathtt{y}\}\ \{\mathsf{ls}(\mathtt{x}, \mathtt{n}, \mathsf{M}, \mathsf{M}, \mathsf{M})\} \rightsquigarrow \{\mathsf{srtl}(\mathtt{x}, \mathtt{n}, \mathtt{lo}, \mathtt{hi}, \mathsf{M}, \mathsf{M}, \mathsf{M})\}.$$

since the logic does not allow to strengthen an arbitrary linked list to a *sorted* linked list without retaining the prior knowledge. Should we have adopted an alternative approach to read-only annotations [9,13] allowing the caller to retain the full permission of the sorted list, then the postcondition of `length` would *not* contain the list-related part of the heap and would only quantify over the result pointer $\{\mathtt{ret} \mapsto \mathtt{n}\}$, thus leading to the solvable goal below:

$$\{\mathtt{x}, \mathtt{y}\}\,; \{\mathsf{srtl}(\mathtt{x}, \mathtt{n}, \mathtt{lo}, \mathtt{hi}, \mathsf{M}, \mathsf{M}, \mathsf{M})\} \rightsquigarrow \{\mathsf{srtl}(\mathtt{x}, \mathtt{n}, \mathtt{lo}, \mathtt{hi}, \mathsf{M}, \mathsf{M}, \mathsf{M})\}.$$

One straightforward way for BoSSL to cope with this limitation is to simply add a version of `length` annotated with specifications that cater to `srtl`.

*Overcoming the limitations.* While the "caller keeps the permission" kind of approach would buy us flexible aliasing and calls with weaker specifications, it would compromise the benefits discussed earlier with respect to the granularity of borrow-polymorphic inductive predicates. One possible solution to gain the best of both worlds would be to design a permission system which allows both borrow-polymorphic inductive predicates as well as read-only modalities to co-exist, where the latter would overwrite the predicate's mixed permissions. In other words, the read-only modality enforces a read-only treatment of the predicate irrespective of its permission arguments, while the permission arguments control the treatment of a mutable predicate. The theoretical implications of such a design choice are left as part of future work.

*Extending read-only specifications to concurrency.* Thus far we have only investigated the synthesis of sequential programs, for which read-only annotations helped to reduce the synthesis cost. Assuming that the synthesiser has the capability to synthesise concurrent programs as well, the borrows annotation mechanism in its current form may not be able to cope with general resource sharing.

This is because a callee which requires read-only permissions to a particular symbolic heap still consumes the entire required symbolic heap from the caller, despite the read-only requirement; hence, there is no space left for sharing. That said, the recently proposed alternative approaches to introduce read-only annotations [9, 13] have no formal support for heap sharing in the presence of concurrency either. To address these challenges, we could adopt a more sophisticated approach based on fractional permissions mechanism [7,8,20,25,30], but this is left as part of future work since it is orthogonal to the current scope.

## 6 Related Work

*Language design.* There is a large body of work on integrating access permissions into practical type systems [5,16,42] (see, *e.g.*, the survey by Clarke *et al.* [10]). One notable such system, which is the closest in its spirit to our proposal, is the borrows type system of the Rust programming language [1] proved safe with RustBelt [22]. Similar to our approach, borrows in Rust are short-lived: in Rust they share the scope with the owner; in our approach they do not escape the scope of a method call. In contrast with our work, Rust's type system carefully manages different references to data by imposing strict sharing constraints, whereas in our approach the treatment of aliasing is taken care of automatically by building on Separation Logic. Moreover, Rust allows read-only borrows to be duplicated, while in the sequential setting of BoSSL this is currently not possible.

Somewhat related to our approach, Naden *et al.* propose a mechanisms for borrowing permissions, albeit integrated as a fundamental part of a type system [31]. Their type system comes equipped with *change permissions* which enforce the borrowing requirements and describe the effects of the borrowing upon return. As a result of treating permissions as first-class values, we do not need to explicitly describe the flow of permissions for each borrow since this is controlled by a mix of the substitution and unification principles.

*Program verification with read-only permissions.* Boyland introduced fractional permissions to statically reason about interference in the presence of *shared-memory concurrency* [8]. A permission $\mathtt{p}$ denotes full resource ownership (i.e. read-write access) when $\mathtt{p} = \mathtt{1}$, while $\mathtt{p} \in (\mathtt{0}, \mathtt{1})$ denotes a partial ownership (i.e. read-only access). To leverage permissions in practice, a system must support two key operations: permission splitting and permission borrowing. Permission splitting (and merging back) follows the split rule: $\mathtt{x} \overset{\mathtt{p}}{\mapsto} \mathtt{a} = \mathtt{x} \overset{\mathtt{p_1}}{\mapsto} \mathtt{a} * \mathtt{x} \overset{\mathtt{p_2}}{\mapsto} \mathtt{a}$, with $\mathtt{p} = \mathtt{p_1} + \mathtt{p_2}$ and $\mathtt{p}, \mathtt{p_1}, \mathtt{p_2} \in (\mathtt{0}, \mathtt{1}]$. Permission borrowing refers to the safe manipulation of permissions: a callee may remove some permissions from the caller, use them temporarily, and give them back upon return.

Though it exists, tool support for fractional permissions is still scarce. Leino and Müller introduced a mechanism for storing fractional permissions in data structures via dedicated access predicates in the Chalice verification tool [27]. To promote generic specifications, Heule *et al.* advanced Chalice with instatiable abstract permissions, allowing automatic fire of the split rule and symbolic borrowing [20]. VeriFast [21] is guided by contracts written in Separation Logic and assumes the existence of lemmas to cater for permission splitting. Viper [30]

is an intermediate language which supports various permission models, including abstract fractional permissions [4,43]. Similar to CHALICE, the permissions are attached to memory locations using an accessibility predicate. To reason about it, VIPER uses permission-aware assertions and assumptions, which correspond in our approach to the unification and the substitution operations, respectively. Like VIPER, we enhance the basic memory constructors, that is blocks and points-to, to account for permissions, but in contrast, the CALL rule in our approach is standard, *i.e.*, *not* permission-aware.

These tools, along with others [3,18], offer strong correctness guarantees in the presence of resource sharing. However, there is a class of problems, namely those involving predicates with mixed permissions, whose guarantees are weakened due to the general fractional permissions model behind these tools. We next exemplify this class of problems in a sequential setting. We start by considering a method which resets the values stored in a linked-list while maintaining its shape ($\mathtt{p} < 1$ below is to enforce the immutable shape):

$$\{\mathtt{p} < 1;\ \mathsf{ls}(\mathtt{x}, \mathtt{S})[1, \mathtt{p}]\}\ \texttt{void reset(loc x)}\ \{\mathsf{ls}(\mathtt{x}, \{0\})[1, \mathtt{p}]\}.$$

Assume a call to this method, namely `reset(y)`. The caller has full permission over the entire list passed as argument, that is $\mathsf{ls}(\mathtt{y}, \mathtt{B})[1, 1]$. This attempt leads to two issues. The first has to do with splitting the payload's permission (before the call) such that it matches the callee's postcondition. To be able to modify the list's payload, the callee must get the payload's full ownership, hence the caller should retain 0: $\mathsf{ls}(\mathtt{y}, \mathtt{B})[1, 1] = \mathsf{ls}(\mathtt{y}, \mathtt{B})[0, 1/2] * \mathsf{ls}(\mathtt{y}, \mathtt{B})[1, 1/2]$. But 0 is not a valid fractional permission. The second issue surfaces while attempting to merge the permissions after the call: $\mathsf{ls}(\mathtt{y}, \mathtt{B})[0, 1/2] * \mathsf{ls}(\mathtt{y}, \{0\})[1, 1/2]$ is invalid since the two instances of `ls` have incompatible arguments (namely $\mathtt{B}$ and $\{0\}$). To avoid such problems, BoSSL abandons the split rule and instead always manipulates full ownership of resources, hence it does not use fractions. This compromise, along with the support for symbolic borrows, allows ROBoSuSLik to guarantee read-only-ness in a sequential setting while avoiding the aforementioned issues. More investigations are needed in order to lift this result to concurrency reasoning. Another feature which distinguishes the current work from those based on fractional permissions, is the support for permissions as parameters of the predicate, which in turn supports the definition of predicates with mixed permissions.

Immutable specifications on top of Separation Logic have also been studied by David and Chin [13]. Unlike our approach which treats borrows as polymorphic variables that rely on the basic concept of substitution, their annotation mechanism comprises only constants and requires a specially tailored entailment on top of enhanced proof rules. Since callers retain the heap ownership upon calling a method with read-only requirements, their machinery supports flexible aliasing and cut-point preservation—features that we could not find a good use for in the context of program synthesis. An attempt to extend David and Chin's work by adding support for predicates with mixed permissions [11] suffers from significant annotation overhead. Specifically, it employs a mix of mutable, immutable, and *absent* permissions, so that each mutable heaplet in the precondition requires a corresponding matching heaplet annotated with absent in the postcondition.

Charguéraud and Pottier [9] extended Separation Logic with RO assertions that can be freely duplicated or discarded. Their approach creates lexically-scoped copies of the RO-permissions before emitting a call, which, in turn, involves discarding the corresponding heap from the postcondition to guarantee a sound RO-modality. Adapting this modality to program synthesis guided by pre- and postconditions would require a completely new system of deductive synthesis since most of the rules in SSL are not designed to handle the *d*iscardable RO-heaps. In contrast, BoSSL supports permission-parametric predicates (*e.g.*, (9)) requiring only minimal adjustments to its host logic, *i.e.*, SSL.

*Program synthesis.* BoSSL continues a long line of work on program synthesis from formal specifications [26,36,40,41,44] and in particular, *deductive synthesis* [14,23,29,33,34], which can be characterised as search in the space of *proofs* of program correctness (rather than in the space of programs). Most directly BoSSL builds upon our prior work on SSL [34] and enhances its specification language with read-only annotations. In that sense, the present work is also related to various approaches that use *non-functional* specifications as input to synthesis. It is common to use *syntactic* non-functional specifications, such as grammars [2], sketches [36,40], or restrictions on the number of times a component can be used [19]. More recent work has explored *semantic* non-functional specifications, including type annotations for resource consumption [24] and security/privacy [17,35,39]. This research direction is promising because (a) annotations often enable the programmer to express a strong specification concisely, and (b) checking annotations is often more compositional (*i.e.*, fails faster) than checking functional specifications, which makes synthesis more efficient. In the present work we have demonstrated that both of these benefits of non-functional specifications also hold for the read-only annotations of BoSSL.

### 7 Conclusion

In this work, we have advanced the state of the art in program synthesis by highlighting the benefits of guiding the synthesis process with information about memory access permissions. We have designed the logic BoSSL and implemented the tool ROBoSuSLik, showing that a minimalistic discipline for read-only permissions already brings significant improvements *wrt.* the performance and robustness of the synthesiser, as well as *wrt.* the quality of its generated programs.

*Acknowledgements.* We thank Alexander J. Summers, Cristina David, Olivier Danvy, and Peter O'Hearn for their comments on the prelimiary versions of the paper. We are very grateful to the ESOP 2020 reviewers for their detailed feedback, which helped to conduct a more adequate comparison with related approaches and, thus, better frame the conceptual contributions of this work.

Nadia Polikarpova's research was supported by NSF grant 1911149. Amy Zhu's research internship and stay in Singapore during the Summer 2019 was supported by Ilya Sergey's start-up grant at Yale-NUS College, and made possible thanks to UBC Science Co-op Program.
